# Drug development for the treatment of onchocerciasis: Population pharmacokinetic and adverse events modeling of emodepside

**DOI:** 10.1371/journal.pntd.0010219

**Published:** 2022-03-10

**Authors:** Frauke Assmus, Richard M. Hoglund, Frédéric Monnot, Sabine Specht, Ivan Scandale, Joel Tarning

**Affiliations:** 1 Mahidol Oxford Tropical Medicine Research Unit, Faculty of Tropical Medicine, Mahidol University, Bangkok, Thailand; 2 Centre for Tropical Medicine and Global Health, Nuffield Department of Medicine, University of Oxford, Oxford, United Kingdom; 3 Drugs for Neglected Disease *initiative*, Geneva, Switzerland; National Institute of Allergy and Infectious Diseases, UNITED STATES

## Abstract

**Background:**

To accelerate the progress towards onchocerciasis elimination, a macrofilaricidal drug that kills the adult parasite is urgently needed. Emodepside has shown macrofilaricidal activity against a variety of nematodes and is currently under clinical development for the treatment of onchocerciasis. The aims of this study were i) to characterize the population pharmacokinetic properties of emodepside, ii) to link its exposure to adverse events in healthy volunteers, and iii) to propose an optimized dosing regimen for a planned phase II study in onchocerciasis patients.

**Methodology / Principal findings:**

Plasma concentration-time profiles and adverse event data were obtained from 142 subjects enrolled in three phase I studies, including a single-dose, and a multiple-dose, dose-escalation study as well as a relative bioavailability study. Nonlinear mixed-effects modeling was used to evaluate the population pharmacokinetic properties of emodepside. Logistic regression modeling was used to link exposure to drug-related treatment-emergent adverse events (TEAEs). Emodepside pharmacokinetics were well described by a transit-absorption model, followed by a 3-compartment disposition model. Body weight was included as an allometric function and both food and formulation had a significant impact on absorption rate and relative bioavailability. All drug-related TEAEs were transient, and mild or moderate in severity. An increase in peak plasma concentration was associated with an increase in the odds of experiencing a drug-related TEAE of interest.

**Conclusions/Significance:**

Pharmacokinetic modeling and simulation was used to derive an optimized, body weight-based dosing regimen, which allows for achievement of extended emodepside exposures above target concentrations while maintaining acceptable tolerability margins.

## Author summary

Onchocerciasis (‘River Blindness’), is a worm infection common in sub-Saharan Africa. More than 20 million people are suffering from the disease which can lead to disfiguring skin disease, visual impairment and permanent blindness. The currently recommended treatment is ivermectin, which kills the juvenile worms and reduces the severity of the symptoms, but fails to kill the adult worm. As a consequence, the treatment needs to be repeated for 10–15 years (life span of the adult worm), imposing a large burden on patients and communities. Hence, there is an urgent need for a new, safe and short-course drug that kills the adult worm and offers a rapid cure. Emodepside is a promising drug candidate which has recently been administered to healthy volunteers for the first time. In this study, we characterized the relationship between dose, systemic exposure in human and the probability of experiencing an adverse event. Modeling and simulation were used to propose a short-course dosing regimen which balances risks and benefits in order to achieve efficacy while maintaining safety. Our results support the further clinical development of emodepside thus contributing to onchocerciasis elimination efforts.

## Introduction

Onchocerciasis (‘River Blindness’) is a parasitic infection caused by the filarial worm *Onchocerca volvulus*, which is transmitted through repeated bites of infected blackflies (*ssp*. *Similium*). The Global Burden of Disease Study estimated a prevalence of 20.9 million cases in 2017, with more than 99% thereof living in 31 countries in sub-Saharan Africa [1], and an estimated 205 million being at risk of infection [2]. In the human host, adult worms can live up to approximately 15 years in subcutaneous nodules [3], releasing millions of larvae (microfilariae) into the surrounding connective tissue, skin and eyes [4]. Clinical manifestations are mainly associated with the human’s immune response to dying microfilariae [5], leading to disfiguring dermatitis, severe itching, and in many cases, visual impairment, or permanent blindness [1].

Global efforts to control onchocerciasis as a public health problem have been ongoing for more than four decades [6–8] and have largely reduced the morbidity and burden of disease in the African and American region [2]. Four of six endemic countries in the American region have been verified as free of onchocerciasis. Moreover, the World Health Organization (WHO) has targeted onchocerciasis for elimination in the majority of endemic African countries by 2025. The cornerstone of control and elimination programs is ivermectin, which kills the microfilariae, and temporarily sterilizes the adult parasite. However, ivermectin lacks macrofilaricidal activity and hence, treatments need to be repeated once or twice per year for over a decade (during the life span of the adult worm), making implementation and widespread coverage extremely difficult. Elimination efforts are further complicated in areas co-endemic with Loa Loa due to the risk of severe side effects to ivermectin in individuals carrying high loads of Loa Loa microfilariae [9,10]. In addition, sub-optimal responses to ivermectin have raised concerns about the development of drug resistance after many years of mass drug administration (MDA) [11].

On the road from control to elimination of onchocerciasis, alternative treatment strategies are thus required [12–16] and there is an urgent need for a safe drug that kills the adult worm (macrofilaricide) and allows for a short-course curative treatment of onchocerciasis, ideally also in patients co-infected with Loa Loa [17]. Emodepside (BAY 44 4400) is a promising macrofilaricide [18,19], and is registered as a veterinary drug for the treatment of gastrointestinal helminths in dogs and cats. Beyond this spectrum of action, emodepside has shown anthelminthic activity against a wide range of other nematodes in various animal hosts, including chicken, mice, rats, gerbil, sheep, cattle and horses [18,20]. Recently, the *in vitro* filaricidal activity spectrum of emodepside has been evaluated for a range of filarial nematodes (e.g. *Litomosoides sigmodontis*, *Onchoerca gutturosa*, *Onchocerca lienalis*) used as model parasites for human filariasis [21]. Dose-dependent inhibition of worm motility by emodepside after 3–5 days of incubation was observed across all tested filarial species and stages. Moreover, embryotoxic, microfilaricidal and partial macrofilaricidal activity of emodepside has been demonstrated in cattle infected with *Onchocerca ochengi*, the closest known relative of the human parasite [22]. Importantly, emodepside was also shown to be fully effective against nematodes in sheep and cattle that are resistant to other classes of anthelminthic drugs [23]. The mode of action is not entirely understood, but involvement of the calcium-activated potassium channel (SLO-1) and the latrophilin (LAT-1) receptor have been discussed [18,24,25], leading to inhibition of pharyngeal pumping, paralysis, and ultimately the death of the nematode.

Currently, emodepside is under clinical development for the treatment of onchocerciasis, following a repurposing strategy and a collaboration agreement between DNDi and Bayer AG [26]. The safety, pharmacokinetics and relative bioavailability of emodepside after oral dosing in healthy male subjects have been evaluated in three phase I clinical trials, including a First-in-Human study [27]. Briefly, emodepside showed a favorable pharmacokinetic profile with rapid absorption under fasting conditions, rapid initial distribution and slow terminal elimination. The long terminal half-life (> 20 days) was expected to be advantageous in maintaining patient exposure to pharmacodynamically active drug levels [27]. No important safety risks have been identified for emodepside to date. Only mild to moderate non-serious drug-related treatment-emergent adverse events (TEAEs) were reported in all three studies. Non-compartmental analysis and descriptive statistics were applied to describe the pharmacokinetics and safety of emodepside in the individual trials. However, no pharmacokinetic-pharmacodynamic (PK/PD) modeling analysis for emodepside has been performed hitherto. In this work, we present a population pharmacokinetic-pharmacodynamic (PK/PD) analysis, based on pooled data from the three phase I clinical trials, using a nonlinear mixed-effects modeling approach. The aims of this study were i) to comprehensively characterize the population pharmacokinetic properties of emodepside and ii) to explore the relationship between emodepside plasma exposure and drug-related adverse events in healthy volunteers. Effects of emodepside formulation, food, dosing regimen and patient-specific characteristics were investigated in order to explain sources of variability in PK parameters. Results from the PK/PD modeling were used to guide the selection of a dosing regimen for a planned phase II study in onchocerciasis patients in sub-Saharan Africa.

## Methods

### Study design and ethical approval

Pharmacokinetic data was pooled from three individual phase I studies [27], including a single ascending dose (SAD) study, a multiple ascending dose (MAD) study, and a relative bioavailability (RelBA) study. A total of 153 subjects received emodepside in these trials as follows: i) SAD study (n = 58): single dose of 1, 2.5, 5, 10, 20 or 40 mg, as oral liquid service formulation (LSF) solution or immediate-release tablet X; ii) MAD study (n = 18): 5 mg once daily, 10 mg once daily, and 10 mg twice daily for 10 days, as oral LSF solution; iii) RelBA study (n = 77): single dose of 5 or 10 mg, as amorphous solid dispersion (ASD) tablet formulation A and B. Dense blood samples were collected in all volunteers (sampling schemes summarized in **Table 1**). Eleven subjects in the SAD study received emodepside as immediate-release tablet X and were omitted from the analysis as this tablet formulation was not carried forward for clinical development due to insufficient plasma exposure. One subject in the SAD study (Cohort 1) had his treatment discontinued (0.1 mg emodepside LSF), and was subsequently withdrawn from the study owing to a pre-treatment adverse event. This subject was likewise excluded from the present analysis. More details about study designs are provided in [27].

**Table 1:** Summary of clinical studies contributing to the pooled pharmacokinetic analysis.

Characteristic	SAD	MAD	RelBA
**Study description**	Blinded, Randomized, Placebo-Controlled, Parallel-Group, Single-Dose, Dose-Escalation Study to assess the PK after oral dosing (incl.food effects and relative bioavailability)	Single-Blind, Randomized, Placebo- Controlled, Parallel-Group, Multiple-Dose-Escalation Study to assess the PK after multiple doses of an oral LSF solution	Open-Label, Randomized, Parallel-Group, Relative Bioavailability Study to compare two new ASDS- tablet formulations to an LSF solution
**No. of subjects**	47 (58 before exclusion of tablet X)	18	77
**ClinicalTrials.gov identifier**	NCT02661178	NCT03383614	NCT03383523
**Dose, formulation and food state**	Single 1, 2.5,5,10, 20 or 40 mg dose; LSF solution or tablet X (excluded)^a^ Fasted or fed	Multiple 5 or 10 mg dose/ 10 days, once or twice daily; LSF solution Fasted	Single 5 or 10 mg dose; LSF solution or immediate-release tablet A or B; Fasted or fed
	Cohort 1,2,3,5,6,8: 1, 2.5,5,10, 20 or 40 mg dose, LSF solution, fasted Cohort 4,7: 5 or 20mg, tablet X, fasted^a^ Cohort 9: 10mg, LSF solution, fed Cohort 10: 40mg, LSF solution, fasted^b^	Cohort 1: 5mg, once daily Cohort2: 10 mg, once daily Cohort 3: 10 mg, twice daily	Cohort 1: 5mg, oral solution, fasted Cohort 2,3: 5mg, tablet A or B, fasted Cohort 4,5: 5mg, tablet A or B, fed Cohort 6,7: 10mg, tablet A or B, fasted
**Sampling scheme (venous plasma)**	Predose, 0.5, 1, 1.5, 2, 2.5, 3, 4, 6, 8, 12, 24, 36, 48, 72, 96, 120, 144 and 168 h postdose, + Follow-up (~ 340 h postdose); Additional samples at 240, 432, 504h postdose (only for 4/10 cohorts)	Day 0: Predose, 0.25, 0.5, 1, 1.5, 2, 2.5, 3, 4, 6, 8, 12, 15h after first dose, Day 1–8: before the morning dose Day 9: predose, 0.25, 0.5, 1, 1.5, 2, 2.5, 3, 4, 6, 8, 12, 15, 24, 36, 48, 72, 96 and 120 h postdose + Follow-up (17,20,23,27 and 30 days after first dose)	Predose, 0.25, 0.5, 1, 1.5, 2, 2.5, 3, 4, 6, 8, 12, 24, 36, 48, 72 h postode, + Follow-up (120 and 168h^c^ postdose)
**Sampling scheme (DBS)**	Predose, 0.5, 1, 6, 8, 12, 24, 36, 48, 72, 96, 120, 144, 168, 240, 336 and 432 h postdose	Day 0: 0.25, 0.5, 1, 1.5, 2, 2.5, 3, 4, 6, 8, 12, and 15 h after first dose Day 1–8: before the morning dose Day 9: before and 24h after the morning dose	-

**Abbreviations:** SAD, single ascending dose study; MAD, multiple ascending dose study; RelBA, relative bioavailability study; PK, pharmacokinetic; LSF, Liquid servive formulation (LSF); ASD-tablet, amorphous solid dispersion tablet formualtion.

^a^subjects administered the tablet formulation from the SAD study were excluded (tablet X not carried forward for clinical development).

^b^Cohort with additonal ophthalmology assessments.

^c^Blood samples for 2/77 subjets was considerably delayed (240h and 350h prostdose).

Briefly, all trials were randomized, parallel-group studies carried out between 2016 and 2018 in a single site specialized in phase I studies (Hammersmith Medicines Research (HMR) Limited, London, United Kingdom). The three studies were approved by local research ethics committees in the United Kingdom and were conducted in accordance with the Declaration of Helsinki and the International Conference on Harmonisation E6 Guideline for Good Clinical Practice. Written informed consent was obtained from all subjects before undertaking any study-related procedures.

### Investigational products

Emodepside was supplied as a LSF solution and three different types of immediate-release tablet formulations. The LSF was a 0.1% (w/v) solution containing 1 mg emodepside/mL. The conventional immediate-release tablet formulation (tablet X, supplied in the SAD study) contained emodespide in crystalline form and was excluded from the present analysis. Pharmacokinetic data from two new tablet formulations evaluated in the RelBA study and referred to as ASD-tablet A and ASD-tablet B were included. Compared to the previous tablet formulation X, these new formulations contained an amorphous dispersion of emodepside formed in granules with either the polymer hypromellose acetate succinate (emodepside coated ASD-tablet A) or the polymer copovidone (emodepside coated ASD-tablet B), in order to increase the apparent solubility and bioavailability of emodepside. Additional excipients (microcrystalline cellulose, croscarmellose sodium, macrogol (15)-hydroxystearate, magnesium stearate) were identical between ASD-tablet A and B. The LSF, tablets and matching placebos were developed and manufactured by Bayer AG. Manufacturing, packaging, quality control and preparation of clinical supplies complied with Good Manufacturing Practice.

### Study subjects

Participants were eligible for the study if they were aged 18–55 years (18–45 years in the RelBA study), with a body mass index (BMI) in the range of 18 to 30.1 kg/m^2^ at screening, and deemed healthy on the basis of a clinical history, physical examination, electrocardiogram (ECG), vital signs, and laboratory tests of blood and urine.

Subjects with a history of residing for ≥ 6 continuous months within the last 3 years in regions with endemic parasitic infections were excluded. Further key exclusion criteria were presence or history of drug or alcohol abuse, frequent smoking, blood loss > 400 mL, recent use of prescription medication and use of dietary supplements or herbal remedies known to be relevant substrates of CYP3A4 and/or P-glycoprotein. Finally, subjects were excluded if they showed severe allergies, hypersensitivity to the investigational drug or drug formulation or if they had a blood pressure and heart rate at the screening outside of the ranges (90–140 mm Hg systolic, 60–90 mm Hg diastolic, heart rate 40–100 beats/min).

### Blood collection and quantification

Blood samples (5mL) for the determination of emodepside concentrations in plasma were collected pre-dose and at time points indicated in **Table 1**. The exact sample times were recorded and used for the population pharmacokinetic modeling. Plasma samples were shipped on dry ice to Analytical Services International (ASI, London UK) for drug quantification. Extra blood samples (≤ 1 mL) were taken from selected cohorts for the evaluation of a dried blood spots (DBS) method (SAD study, cohort 10, n = 6 subjects and MAD study, cohort 1, n = 6 subjects). Whole venous blood spots were collected onto DMPK-B cards, dried, and assayed for emodespide at Swiss BioQuant AG, Reinach, Switzerland.

Plasma and DBS samples were analyzed using validated liquid chromatography—tandem mass spectrometry (HPLC-MS/MS) methods (S1 Table, Supplementary Information). Deuterated emodepside-D16 was used as the internal standard. Total assay coefficients of variation (CV) for emodepside during analysis were within the acceptable limits and did not exceed 5% (plasma samples) and 10% (DBS samples) at all quality control levels. The lower limit of quantification (LLOQ) was 1 ng/mL for both plasma and DBS samples.

### Population pharmacokinetic analysis

#### (i) Model development

Emodepside venous plasma and DBS concentrations were pooled across three phase I studies from 142 subjects, and were transformed into their natural logarithm. The concentration-time profiles were modeled simultaneously using nonlinear mixed-effects modeling and the first-order conditional estimation method with interactions (FOCE-I) in NONMEM, version 7.4.3 (Icon Development Solution, Ellicott City, MD, USA). The model building process and model diagnostics were facilitated by using Pirana version 2.9.8, Pearl-speaks-NONMEM (PsN; version 3.5.3), R version 3.6 and the R package Xpose version 4.0.

Concentrations below the LLOQ were omitted since only 4.66% of all samples were reported below the LLOQ. A population based, linear conversion factor was estimated to account for systematic differences between concentration measurements in different sampling matrices (i.e., venous plasma and DBS).

One-, two-, three- and four- compartment disposition models were evaluated to characterize the distribution of emodepside, based on a first order-absorption model. Following the selection of the best structural disposition model, different absorption models were explored, including first-order absorption models with and without lag time, and transit compartment absorption models. Between 1 and 10 transit compartments were tested, with absorption rate (k_a_) and rate constants between transit compartments (k_tr_) either assumed to be equal or estimated separately. Mean absorption transit time (MTT) was estimated and used to calculate k_tr_ (Eq 1)

$$K_{tr}=\frac{\left(1+number\;of\;transit\;compartments\right)}{MTT}$$ (1)

Inter-individual variability (IIV) in all pharmacokinetic parameters was modeled using an exponential error model, according to (Eq 2):

$${ϴ}_{i}=ϴ\times e^{n_{i,ϴ}}$$ (2)

where ϴ_i_ is the individually estimated parameter for the i^th^ subject, ϴ is the population mean parameter estimate, and η_i,ϴ_ is the IIV for parameter ϴ, drawn from a normal distribution with a zero mean and ω^2^ variance. Due to the limited number of subjects with DBS data, no IIV was allowed on the venous plasma-to-DBS conversion factor. Relative bioavailability (F) was fixed to unity for the population, but IIV was evaluated in this parameter to account for the high variability in absorption after administration of different emodepside formulations. Regarding pharmacokinetic data from the MAD study, also inter-occasion variability (IOV) was investigated on absorption parameters (MTT, and F) according to (Eq 3):

$${ϴ}_{i,j}=ϴ\times e^{n_{i,ϴ}+\kappa_{j,ϴ}}$$ (3)

where ϴ_i,j_ is the individually estimated parameter for the i^th^ subject at the j^th^ occasion, and κ_j,ϴ_ is the IOV for parameter ϴ. Two occasions were defined (occasion 1: ≤6 days, occasion 2: ≥7 days), taking into consideration that rich pharmacokinetic data was only available on the first and the tenth day of dosing (**Table 1**).

Estimated IIV below 10% was fixed to zero. The unexplained residual variability was modeled separately for venous plasma and DBS concentrations, with an additive error model for log-transformed emodepside concentrations (essentially equivalent to an exponential residual error on an arithmetic scale).

The influence of covariates on pharmacokinetic model parameters was evaluated based on biological plausibility, statistical significance and model performance. Body weight was taken into account by allometric scaling (standardized to a body weight of 75 kg), according to (Eq 4):

$${ϴ}_{i}=ϴ\times e^{n_{i,ϴ}}\times {\left(\frac{{BW}_{i}}{75}\right)}^{n}$$ (4)

where BW_i_, represents the individual body weight of the i^th^ subject. The exponent *n* was fixed to 0.75 for all clearance parameters, and to 1 for all volume of distribution parameters. Various additional biopharmaceutical (formulation, food, dose) and subject characteristics (age, liver/kidney function / haematology markers) were evaluated as potential covariates. Individual creatinine clearance was calculated from creatinine data using the Cockcroft and Gault equation. Redundant covariates showing high cross correlation in bivariate linear regression analysis (r^2^ > 0.5) were excluded, along with covariates with little variability (e.g. smoking) or lack of unambiguous interpretability. Preselected covariates (formulation, food, dose [dose per day, dose per kg body weight per day], age, alanine aminotransferase [ALAT], alkaline phosphatase [ALP], aspartate aminotransferase [AST], bilirubin (total and conjugated), creatinine clearance [CrCL], haemtocrit ratio, gamma–glutamyl transferase [GGT], lactate dehydrogenase [LDH], urea) were further evaluated by a step-wise covariate modeling approach. In the forward step, covariates were included at a statistical significance level of p = 0.05, followed by a more stringent backward elimination step (p = 0.001). Formulation and food effects on absorption parameters (MTT, F) were evaluated manually (as categorical variables) using the base model, and significant covariates were carried forward to the stepwise covariate model evaluation. All additional covariates (all continuous) were evaluated using the stepwise covariate model functionality in PsN. Linear, exponential, and power parameter-covariate relationships, centered on their median values for the population, were explored.

#### (ii) Model evaluation

Discrimination between two hierarchical models was based on a comparison of objective function value (OFV) values (proportional to -2 times the log-likelihood of the data). The difference in OFV (ΔOFV) is equivalent to a likelihood ratio test (two-sided), with ΔOFV > 3.84 and > 10.83 being considered statistically significant at a p value of < 0.05 and < 0.001, respectively, when comparing two nested models with one degree of freedom difference. Basic goodness-of-fit diagnostics were used to evaluate the descriptive performance of the model and identify potential systematic errors and model misspecification. Moreover, shrinkage values were calculated to evaluate the reliability of the individual parameter estimates and goodness-of fit diagnostics. Bootstrapping (n = 1,000 resampled bootstrap datasets) was performed to obtain parameter precision estimates, i.e. RSEs and CIs. The final model was also evaluated by a prediction-corrected numeric and visual predictive check (n = 2,000 simulations) to evaluate the predictive power of the model. To this purpose, the 5th, 50th, and 95th percentiles of the observed data were overlaid with the 95% confidence intervals of simulations for the same percentiles.

### Safety analysis

For a detailed safety analysis for emodepside, based on the entire safety population (pharmacokinetic sample and placebo arms) of the phase I clinical trials the reader is referred to the recently published article by Gillon *et al*. [27]. In the present study, the safety analysis and exposure–adverse events analysis was performed on all subjects whom were included in the population pharmacokinetic analysis (n = 142), i.e. all subjects who received at least 1 dose of the study drug as LSF solution, ASD-tablet A or ASD-tablet B. Safety was assessed in terms of incidence of a drug-related TEAE (as assessed by the investigator) and reported as Medical Dictionary for Regulatory Activities (MedDRA) code. A TEAE was defined as an event that emerged during treatment and having been absent pretreatment, or that worsened relative to the pretreatment state.

### Exposure–adverse events analysis

The relationship between drug exposure and adverse events was evaluated by binary logistic regression modeling in R, according to (Eq 5):

$$logit(p)=p/(1-p)=\beta_{0}+\beta_{1}x+\varepsilon ,$$ (5)

where *p* is the probability and logit (*p*) the log odds of experiencing a drug-related TEAE of interest, β_0_ is the intercept and β_1_ the coefficient of the predictor variable x; ε denotes the residual error. A drug-related TEAE of interest was defined as eye disorder or nervous system disorder. All drug-related TEAEs of interest were modeled, irrespective of whether or not unspecific adverse events occurred before the occurrence of a drug-related TEAE of interest. In addition, separate logistic exposure-response regression models were developed for the occurrence of drug-related, treatment-emergent eye disorders and nervous system disorders. Different exposure variables (derived from the final pharmacokinetic model) were investigated as predictors of adverse events, namely individual maximum plasma concentrations (C_max_) and areas under the concentration-time curve (AUC_∞_), as well as daily dose and cumulative dose (continuous variables).

The models were compared on the basis of the Akaike information criterion (AIC), McFadden’s Pseudo R^2^, p values of the β_1_ coefficients and accuracy. In addition, the area under the Receiver Operating Characteristics (ROC) curve was used as a selection criterion. Demographic factors (bivariate: drinker/no drinker, smoker/non-smoker, normal BMI/obese, categorical: age groups) were investigated as covariates in multivariate logistic regression models and statistical significance was assessed using the log likelihood-ratio test.

### Simulations of dosing scenarios

Stochastic simulations based on the final population pharmacokinetic model were performed in NONMEM to evaluate different dosing regimens. Simulations were performed for emodepside after administration as ASD-tablet formulation B in a fasted state (i.e. the suggested route of administration for the phase II study). C_max_ values and total time above the putative minimum target concentration (C_target_ = 100 ng/mL) were simulated for 1,000 adults per body weight group, covering a range of body weights (40–150 kg), taking covariate effects and variability into account. Antiparasitic activity of emodepside *in vivo* was previously evaluated in an *O*.*ochengi*-infected cattle model [22] and an *L*.*sigmodontis*-infected rat model [28]. In the *O*.*ochengi* cattle model, a pronounced reduction in adult worm motility was observed if total plasma concentrations of emodespide were maintained above approximately 100 ng/mL for 7 days. In addition, emodepside showed embryotoxic effects and microfilaricidal activity with prolonged effects on adult female fecundicity [22]. Notably, the effect on adult worm motility and viability as well as microfilariae density in cattle was dose-dependent and multiple doses (0.75mg/kg for 7 days) tended to be more effective compared to a single dose of emodepside [22]. In the *L*.*sigmodontis* rat model, repeated oral dosing (100 mg/kg for 5 consecutive days) yielded a 100% reduction in adult worm burden, while a single dose was not curative [28]. Hence, exposure targets were set to at least 5 days total time above C_target_. It is worth noting that the selected C_target_ is also approximately equal to the total concentration at which *L*.*sigmodontis* microfilariae motility was reduced by 90% *in vitro* (IC_90_ = 0.1 μM, after 4 days of incubation with emodespide [23]).

Drug-related TEAEs of interest were predicted from simulated C_max_ values using the final C_max_-adverse events model. Toxicity thresholds were set to a maximum arbitrary TEAE probability of 50%, which was considered acceptable by the clinical team, given that all drug-related TEAEs of interest were mild (except two subjects experiencing moderate headaches) and transient in nature. Dosing regimens were evaluated based on whether they achieved median total time above C_target_ values higher than the efficacy targets, and median TEAE probabilities lower than the tolerability thresholds for subjects with different body weights. Graphical representations were done in R.

## Results

### Pharmacokinetic data and characterization of the study population

A total of 142 healthy male subjects were included in the pooled population pharmacokinetic analysis, with 47, 18 and 77 subjects contributing pharmacokinetic data from the SAD, MAD and RelBA study, respectively. A summary of study designs is presented in **Table 1** and baseline characteristics of the study participants are provided in **Table 2**. All subjects were healthy, male, White adults (age: 18–54 years).

**Table 2:** Summary of baseline demographic and clinical data for the study populations contributing to the present analysis.

Characteristic	SAD	MAD	RelBiA	Pooled Analysis
Volunteers, n (%)	47 (33.1)	18 (12.7)	77 (54.2)	142 (100)
Age (years)	32 (19–54)	30 (19–43)	32 (18–44)	32 (18–54)
Body weight (kg)	78.2 (59.0–100)	74.1 (54.2–95.2)	80.4 (53.2–105)	79.1 (53.2–105)
BMI (kg/m^2^)	24.1 (19.0–29.2)	22.7 (18.1–27.8)	24.8 (18.9–30.1)	24.3 (18.1–30.1)
Smoker, n (%)	4 (8.51)	4 (22.2)	20 (26.0)	28 (19.7)
Alanine Aminotransferase (IU/L)	20 (7–61)	18 (9–32)	22 (11–57)	21 (7–61)
Alkaline Phosphatase (IU/L)	53 (33–107)	55 (33–81)	51 (33–97)	52 (33–107)
Aspartate Aminotransferase (IU/L)	21 (15–33)	21 (16–28)	23 (16–42)	22 (15–42)
Bilirubin, total (umol/L)	12.5 (5.3–20.5)	12.3 (8.2–26.3)	14.6 (5.0–35.6)	13.3 (5.0–35.6)
Gamma-glutamyl transferase (IU/L)	14 (7–48)	12 (8–22)	15 (7–115)	14 (7–115)
Lactate dehydrogenase (IU/L)	120 (84–194)	113 (90–173)	123 (79–172)	120 (79–194)
Urea (mmol/L)	5.6 (2.6–8.1)	5.4 (3.2–8.2)	4.6 (2.4–7.4)	5.0 (2.4–8.2)
Creatinine Clearance (mL/min)	139 (77–205)	137 (77–167)	148 (89–218)	143 (77–218)
Haematocrit ratio	0.43 (0.37–0.49)	0.43 (0.39–0.47)	0.42 (0.37–0.46)	0.43 (0.37–0.49)

All values are given as median (range, min-max), unless otherwise indicated. All participants were White males. 2/142 subjects (1.41%) were of Hispanic/Latino ethnicity; all other subjects were ‘not Hispanic/Latino’ in ethnicity.

All subjects received an oral dose of emodepside (dose range: 1–40 mg), either in fasted (n = 113) or fed state (n = 29). The majority of participants was administered emodepside as an oral LSF solution (n = 76; 70/76 in a fasted state). The remaining participants received emodepside as two amorphous solid dispersion tablet formulations; 35 subjects (23/35 in a fasted state) received ASD-tablet A (gastroresistant polymer hypromellose acetate succinate) and 31 subjects (20/31 in a fasted state) received ASD-tablet B (gastrosoluble polymer copovidone). Both tablet formulations were immediate release dosage forms with rapid dissolution under *in vitro* test conditions.

In total, 142 subjects contributed to 3,123 blood concentrations for emodepside available for analysis, thereof 2,892 concentrations measured in venous plasma and 231 concentrations measured in DBS. No pharmacokinetic samples (except samples below the LLOQ) were excluded from the final analysis.

### Pharmacokinetic analysis

Venous plasma and DBS emodepside concentrations were modeled simultaneously by using nonlinear mixed-effects modeling. A linear conversion factor was estimated at the population level (DBS concentration = 61.8% of venous plasma concentrations at any given time), describing the systematically lower DBS compared to venous plasma concentrations (**S1 Fig**).

**Fig 1:**
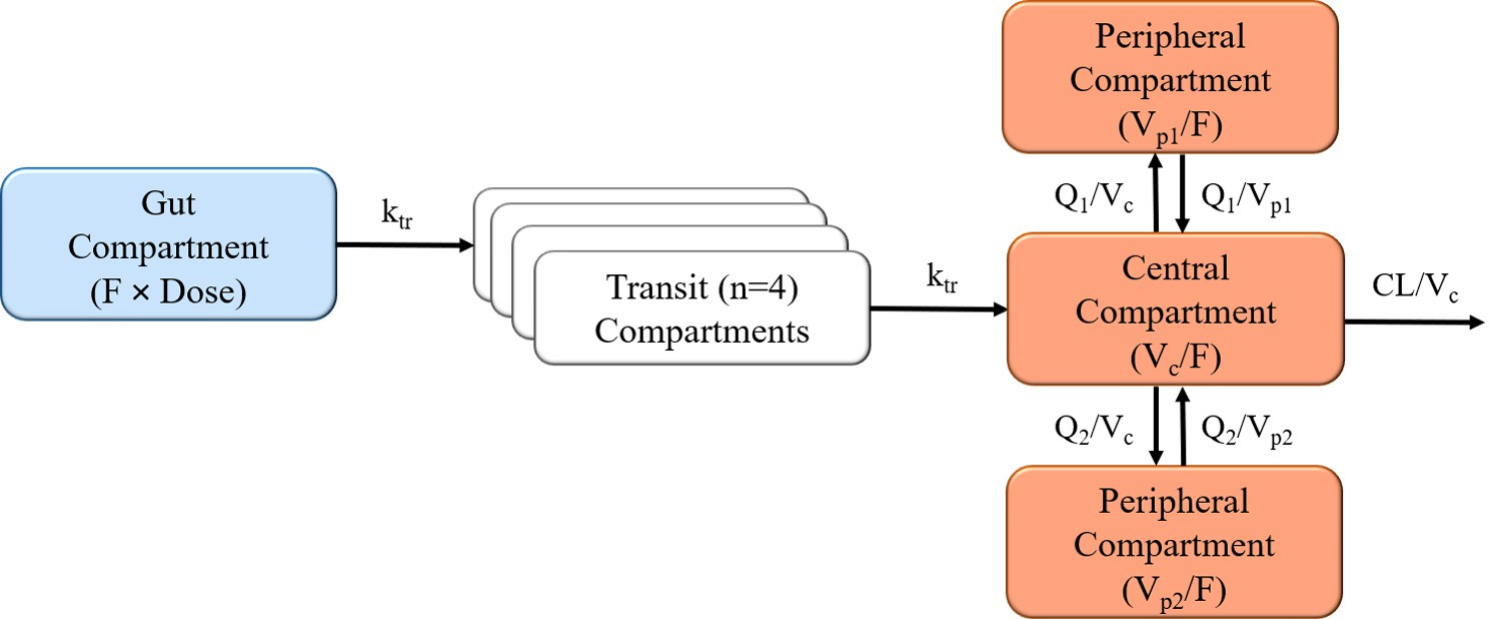
Graphical representation of the final structural model describing the pharmacokinetics of emodepside in healthy volunteers. Absorption from the gut compartment is described by a 4-transit-compartment absorption model, followed by a 3-compartment disposition model. F is the relative oral bioavailability, k_tr_ is the rate constant between absorption compartments, V_c_ is the apparent volume of distribution of the central compartment, V_p_ is the apparent volume of distribution of the peripheral compartments, Q is the inter-compartmental clearance between the central and peripheral compartments, and CL is the apparent elimination clearance.

Briefly, emodepside concentration-time profiles were best described by a transit-compartment absorption model (n = 4), followed by a three-compartment disposition model, with linear elimination from the central compartment. A graphical representation of the final structural model, along with a description of the model parameterization, is presented in **Fig 1**. One- two- and four-compartment disposition models were also tested, but showed systematic model misspecifications, statistical inferiority or low precision in additional pharmacokinetic parameter estimates. Hence, the three-compartment disposition model was carried forward. The addition of F (fixed to unity for the population) with an estimated IIV significantly improved the model fit (ΔOFV = -190) and was thus incorporated into the base model, prior to the exploration of different absorption models.

A four-transit-compartment absorption model gave the best fit to the observed absorption data compared to all other absorption models tested, including a first-order absorption model without (ΔOFV = -3,591) and with a lag time component (ΔOFV = -322). Estimating k_tr_ and k_a_ separately resulted in an unstable model and unreasonable parameter estimates with high variability (710% IIV on k_a_) and low precision; k_tr_ and k_a_ were therefore assumed to be equal.

Body weight was included as an allometric function on all clearance and volume parameters, resulting in a model improvement (ΔOFV = -35.0). Moreover, formulation had a substantial impact on the absorption of emodepside, as indicated by significant reductions in OFVs following the incorporation of formulation effects on MTT (ΔOFV = -133) and F (ΔOFV = - 73). ASD-tablet formulation A and B showed a 243% and 114% longer absorption time as compared to the LSF solution. Bioavailability was estimated to be 68.6% (tablet formulation A) and 80% (tablet formulation B) relative to administration of the LSF solution. ASD-tablet B showed an 11.4% higher F compared to ASD-tablet A and was therefore used for subsequent dose finding simulations.

Food intake had a statistically significant impact on absorption parameters with an estimated 24.4% reduction in F (ΔOFV = -31) and 114% increase in MTT (ΔOFV = -56) when the dose was given together with food. Of the additional covariates tested (age, dose, liver / kidney function and haematology markers), only the effect of dose (expressed as mg per kg body weight per day, dose range: 1–40 mg) on MTT was kept after the backward elimination covariate step (ΔOFV = -11.17). Dose was linearly associated with MTT, with an estimated 105% increase in MTT from the population value for each unit rise in daily emodepside dose (mg/kg).

IOV on absorption parameters was investigated during model development and was found to have a significant influence on MTT (but not F). Separate error models for venous plasma and DBS emodepside concentrations resulted in statistically superior models compared to common error models (p < 0.01), and were therefore implemented throughout the entire model development process. IIV in the apparent volume of distribution of a peripheral compartment (V_p1_) was estimated to be close to zero and was therefore removed from the final model (without affecting the OFV). Important steps in the model building history are summarized in S2 Table.

**Fig 2:**
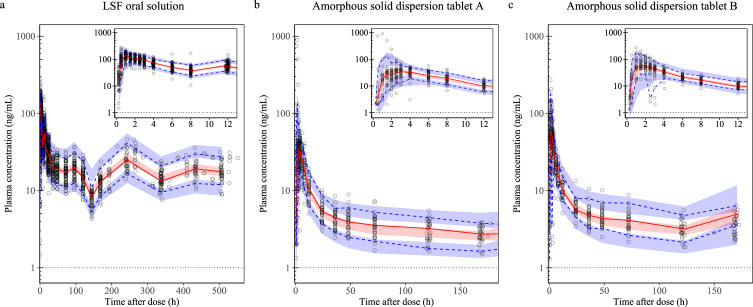
Prediction-corrected visual predictive checks of the final population pharmacokinetic model for emodepside. Strata by formulation of emodepside. **a:** LSF oral solution; **b:** amorphous solid dispersion tablet A; **c:** amorphous solid dispersion tablet B (1 data point at 240 h time after dose was censored). Open circles represent observed plasma emodepside concentrations. The solid red line represents the 50^th^ percentile (median) and the blue dashed lines represent the 5^th^ and 95^th^ percentiles of the observed data. The shaded area represents the 95% confidence interval around the simulated 5^th^, 50^th^, and 95^th^ percentiles. The horizontal dashed line represents the lower limit of quantification of emodepside (1 ng/mL). The inset shows the absorption phase between 0 and 12 h.

Parameter estimates from the final model, along with their standard errors and confidence intervals (CIs), are presented in **Table 3**. The final model described the observed concentration-time profiles well with no major model misspecification (basic goodness-of-fit plots, **S2 Fig**). Bootstrapping indicated a robust pharmacokinetic model with high precision in the estimation of pharmacokinetic parameters and moderate precision in the estimation of covariate effects. Shrinkage was low with respect to epsilon shrinkage (10.9% for venous plasma data and 3.9% for DBS data) as well as eta shrinkage for absorption (MTT 3.3%, F 11.3%) and volume of distribution parameters (V_c_/F 11.6%, V_p1_/F 12.9%). However, eta shrinkage for clearance parameters (CL/F 46.4%; Q_1_/F 40.5%, Q_2_/F 42.8%) and IOV on MTT (~75%) was relatively high. Prediction-corrected visual (**Fig 2**) and numerical predictive checks indicated good predictive performance of the final model for each emodepside formulation investigated. The numerical predictive check after administration of emodepside as LSF oral solution resulted in 1.13% (95% CI 0.99–4.42%) and 1.03% (95% CI 0.99–4.79%) of observations below and above the simulated 95% prediction interval, respectively. Similarly, numeric predictive checks for the ASD-tablet formulations indicated satisfactory prediction accuracies: 1.54% (95% CI 0.39–5.59%) and 2.11% (95% CI 0.42–5.47%) of observations were below the simulated 95% prediction interval for ASD-tablet A and ASD-tablet B, respectively, and 3.66% (95% CI 0.77–5.01%) and 2.32% (95% CI 0.63–5.47) of observations were above the respective thresholds.

**Table 3:** Parameter estimates of the final population pharmacokinetic model of emodepside in healthy male subjects.

Parameter^a^	Population estimate^b^ (%RSE)^c^		Bootstrapping median (95% CI)^c^	IIV/IOV, %CV^b^ (%RSE)^c^	Bootstrapping median (95% CI)^c^
***Pharmacokinetics***					
F	1 *fixed*		-	18.9 (7.8)	18.5 (15.5–21.4)
MTT (h)	0.488 (3.5)		0.489 (0.456–0.521)	38.0 (8.7) / 26.8 (20.7)	36.9 (30.6–43.5) / 26.5 (13.7–36.2)
CL/F (L/h)	1.29 (4.6)		1.29 (1.18–1.40)	21.2 (18.5)	21.2 (12.5–29.1)
V_c_/F (L)	52.4 (3.4)		52.4 (49.2–55.8)	31.1 (9.0)	30.9 (25.0–36.3)
Q_1_/F (L/h)	4.60 (5.1)		4.60 (4.14–5.04)	29.7 (27.1)	30.3 (10.0–45.5)
V_p1_ / F (L)	44.4 (8.7)		45.1 (38.0–53.4)	-	-
Q_2_/F (L/h)	8.45 (2.9)		8.43 (7.97–8.87)	13.1 (26.5)	13.6 (5.2–20.1)
V_p2_/F (L)	647 (4.5)		647 (592–707)	30.8 (9.6)	31.0 (25.6–37.7)
Venous plasma–DBS scaling factor (%)	61.8 (2.9)		61.8 (58.2–65.2)	-	-
σ, venous data	0.0203 (5.7)		0.0202 (0.0160–0.0250)	-	-
σ, capillary data	0.0366 (21.4)		0.0377 (0.0122–0.0792)	-	-
***Covariates***					
Formulation effect on F (%) *reference*: *LSF solution)*	ASD-tablet A	- 31.4 (10.9)	- 31.5 (-37.9 –-24.7)	-	-
	ASD-tablet B	- 20.0 (18.8)	- 19.8 (-26.9 –-12.4)	-	-
Formulation effect on MTT (%) *(reference*: *LSF solution)*	ASD-tablet A	243 (12.2)	241.7 (189–302)	-	-
	ASD-tablet B	124 (14.9)	123.2 (89.2–162)	-	-
Food intake on F *(%) (reference*: *fasted state)*	- 24.4 (17.3)		- 24.1 (- 31.6 –-16.1)	-	-
Food intake on MTT *(%) (reference*: *fasted state)*	114 (22.6)		114.7 (69.0–168)	-	-
Dose on MTT, % per mg /kg/day	105 (22.3)		104.5 (62.9–154)	-	-

^**a**^**Abbreviations:** MTT, mean absorption transit time; CL/F, elimination clearance; V_c_/F, central volume of distribution; Q/F, inter-compartmental clearance between the central and peripheral compartments; V_p_/F, volume of distribution of the peripheral compartments; DBS, dry blood spot; σ, the variance of the residual error; F, relative oral bioavailability; LSF, liquid service formulation; ASD-tablet, amorphous solid dispersion tablet formulation; BW, body weight. Population estimates are given for an adult weighting 75 kg.

^b^ Population mean parameter estimates and inter-individual variability (IIV) calculated by NONMEM. The coefficient of variation (% CV) for the IIV and inter-occasion variability (IOV) was calculated as $100\times \sqrt{e^{\omega^{2}}-1}$.

^c^ Precision of parameter estimates, based on nonparametric bootstrap diagnostics of the final pharmacokinetic model (860 successful runs out of 1,000). Relative standard errors (RSE, %) were calculated as $100\times \frac{standard\;deviation}{mean\;value}$. The 95% confidence intervals (95% CI) were based on the 2.5^th^ –97.5^th^ percentiles of the bootstrap parameter estimates.

Simulated secondary parameter estimates for the LSF solution and tablet formulation A and B are shown in the Supplementary Information (**S3 Table)**. To allow for comparability between emodespide formulations, simulations were based on the final pharmacokinetic model and a standardised dosing regimen (10 mg emodepside, twice daily, for 10 days, for an adult with 75 kg body weight), that corresponds to the dosing regimen proposed for a planned phase II study (see below). In the following, results are only presented for ASD-tablet B, yielding higher exposures as compared to ASD-tablet A. Simulated times to maximum concentration (T_max_) for ASD-tablet B ranged between 2.38 h (90% CI: 1.23–4.54 h, fasted state) and 4.40 h (90% CI: 2.31–8.15 h, fed state). Simulated C_max_ ranged between 258 ng/mL (90% CI: 178–378 ng/mL, fasted state) and 179 ng/mL (123–262 ng/mL, fed state). The simulated terminal elimination half-life of emodepside was 18.4 days (90% CI: 11.0–32.7 days).

### Safety

TEAEs that were considered related to treatment by the investigator were reported in 31 out of 142 subjects (21.8%). All drug-related TEAEs were transient in nature and mild in severity, apart from moderate headaches in two subjects in the highest dose group (40 mg LSF solution). There were no deaths or severe drug-related TEAEs reported. The most frequently affected primary system organ classes for drug-related TEAEs were eye disorders (14.1% [20 subjects]) and nervous system disorders (12.7% [18 subjects]). The most frequently reported drug-related TEAEs (by preferred term) were visual impairment (9.2% [13 subjects]), vision blurred (4.9% [7 subjects]), dizziness (4.9% [7 subjects]) and headache (4.2% [6 subjects]). Other drug-related TEAEs were observed in less than 3% of the study participants (Supplementary Information, **S4 Table**), with similar frequencies in the treatment and placebo groups. Therefore, only eye disorders and nervous system disorders were defined as drug-related TEAEs of interest, which were reported in a total of 27 subjects (19.0%). Eleven of the 27 subjects (41%) experienced both eye disorders and nervous system disorders. Onset of drug-related TEAE of interest ranged from 20 min to 5.5 h post-dose, with most (83%) occurring at 1–3 h post-dose (Supplementary Information, S5 Table). For the majority of drug-related TEAEs of interest, a lag relative to T_max_ was observed (0–2.5 h for most subjects, but up to 4.3 h post-dose). All drug-related TEAEs of interest resolved within 48 h after onset, apart from visual impairment in one subject that resolved after 21 days.

### Exposure–adverse events analysis

**Fig 3:**
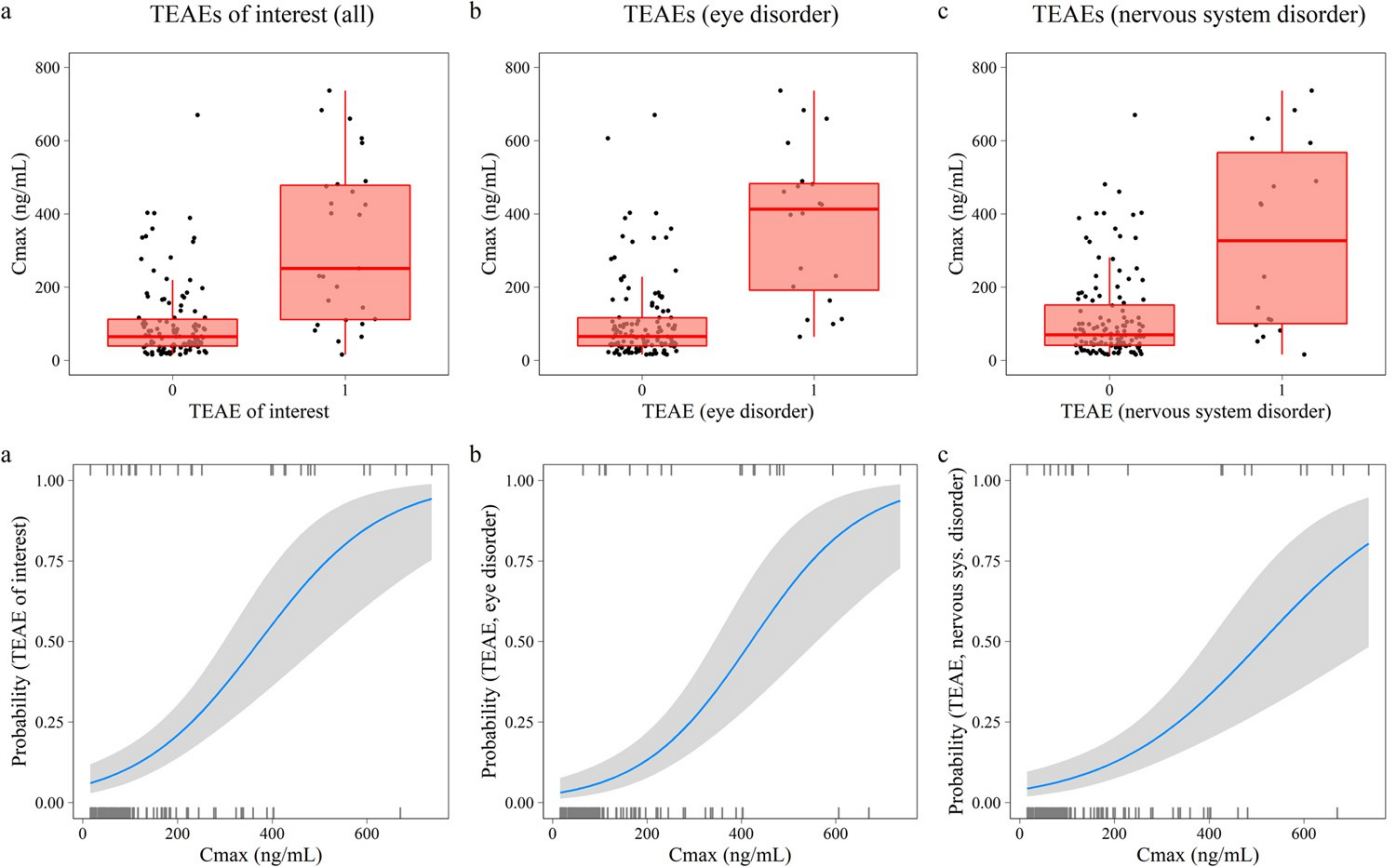
Relationship between emodepside exposure and drug-related TEAEs of interest. Boxplots (upper panel) showing the distribution of C_max_ for study participants without (TEAE of interest = 0) and with (TEAE of interest = 1) occurrence of drug-related TEAE of interest (**a**), as well as eye disorders (**b**) and nervous system disorders separately (**c**). The midline indicates the median, the box corresponds to the interquartile range, and the whiskers extend up to 1.5 times the interquartile range. The lower panel show corresponding predicted probabilities of drug-related TEAE of interest, eye disorder and nervous system disorder, respectively, based on the final logistic C_max_−adverse events regression model. The solid blue line indicates the median and the shaded area the 95% CI around predicted probabilities.

Since all drug-related TEAEs of interest occurred early, a binary logistic regression modeling approach was used to evaluate the relationship between emodepside exposure and adverse events and for subsequent dose finding simulations. Different exposure variables were investigated as predictors of drug-related TEAEs of interest. Non drug-related TEAEs were not included in the logistic regression model (**S1 Text)**. C_max_ was a better TEAE predictor as compared to AUC_∞_, daily dose or cumulative dose, as assessed by various diagnostic criteria (**S6 Table** and **S3 Fig**). The same trend was observed when conducting logistic regression analysis for any type of drug-related TEAE (Supplementary Information, **S7 Table**). Distributions of C_max_ across subjects with and without drug-related TEAE of interest are shown in **Fig 3A** (upper panel). Stratified boxplots for the relationship between C_max_ and eye disorder and nervous system disorder are presented in **Fig 3B and 3C**, respectively.

Binary logistic regression identified a significant correlation between C_max_ and the odds of experiencing a drug-related TEAEs of interest, in both the combined and stratified analysis (**Fig 3A, 3B, 3C** lower panel**).** A 1 ng/mL increase in C_max_ was associated with a 0.77% (95% CI: 0.47%– 1.08%) increase in the odds of experiencing a drug-related TEAE of interest. The association between C_max_ and TEAEs was more pronounced for eye disorders as compared to nervous system disorders, with a 0.86% (95% CI: 0.53% - 1.19%) vs 0.62% (95% CI: 0.35% - 0.91%) odds increase per 1 ng/mL increase in C_max_, respectively. For example, an increase in C_max_ from 300 ng/mL to 400 ng/mL was associated with a median increase in TEAE probability from 36% to 55% (all drug-related TEAEs of interest), 26.3% to 45.6% (only eye disorders), and 21.1% to 33.3% (only nervous system disorders). Further increase in C_max_ to 500 ng/mL was predicted to result in a 73% (all drug related TEAEs of interest), 66% (only eye disorders), and 48% (only nervous system disorder) probability of TEAEs. Multivariate exposure–response analyses, adjusted for demographic factors (age, BMI, smoking, drinking), yielded no significant improvement of the models.

### Emodepside dose finding simulations

Initial dose finding simulations were performed for an adult with 75 kg body weight and administration of emodepside as ASD-tablet B, in the fasted state. The aim was to reach a target concentration of 100 ng/mL and to maximize the time above this target (i.e. C_trough_ > 100 ng/mL, with a minimum of 5 days above this target for at least 50% of the population). Predicted pharmacokinetic profiles suggested that twice daily dosing with 10 mg emodepside for 10 days is required to achieve this (**S4 Fig**). In contrast, once daily dosing with 10 mg emodepside for 7 or 14 days, as well as twice daily dosing with 10 mg emodepside for 7 days failed to reach target exposures.

**Fig 4:**
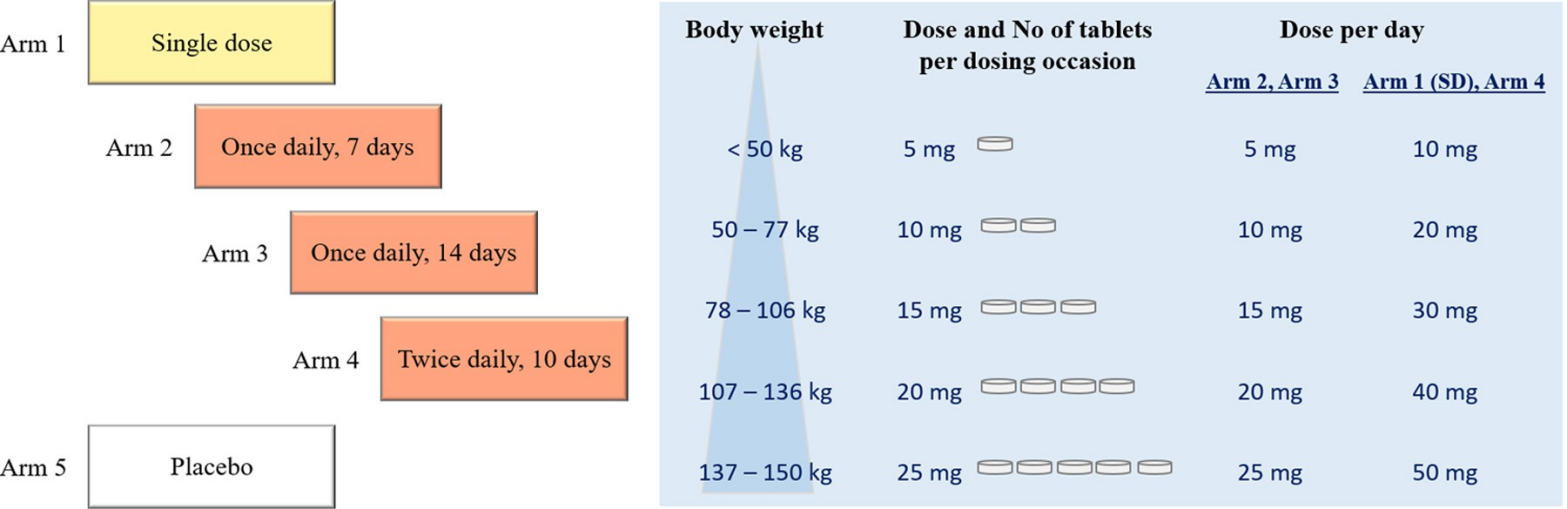
Overview of study arms planned for a phase II clinical trial. Proposed body-weight based dosing regimens for administration of amorphous solid dispersion (ASD) tablet formulation B.

**Fig 5:**
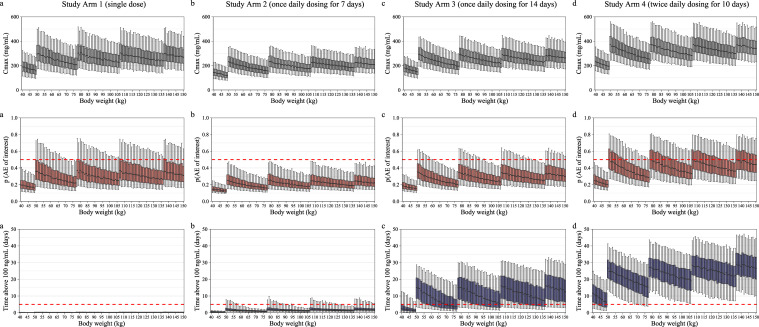
Simulations of emodepside exposure and probability of adverse events for study arm 1–4. Maximum emodepside plasma exposure (C_max_, upper panel), corresponding predicted probability of drug-related TEAE of interest (middle panel) and total time above the target concentration of 100 ng/mL (lower panel), stratified by body weight. The midline of the boxplots indicates the median, the box corresponds to the interquartile range, and the whiskers extend from the 5^th^ to the 95^th^ percentile. The red dotted line in the middle panel indicates a 50% probability threshold for any drug-related TEAE of interest. The red dotted line in the lower panel indicates the minimum number of days (5 days) above the target concentration. Simulations are based on administration of amorphous solid dispersion (ASD) tablet formulation B and the proposed body weight-based dosing regimen for **a:** study arm 1 (single dose of emodespide), **b:** study arm 2 (once daily dosing for 7 days), **c:** study arm 3 (once daily dosing for 14 days) and **d:** study arm 4 (twice daily dosing for 10 days) as described in Fig 4.

Based on this exploratory analysis, four dosing regimens (single dose (study arm 1), once daily dosing for 7 days (study arm 2), once daily dosing for 14 days (study arm 3), and twice daily dosing for 10 days (study arm 4)) were evaluated for a proof-of concept phase II clinical trial, with dose–escalation up to twice daily dosing for 10 days. Both fixed dosing and a proposed body weight-based dosing (**Fig 4**) were simulated. Fixed dosing with 10 mg emodepside per dosing occasion resulted in unreasonably high probabilities of TEAEs of interest, especially for subjects with body weights < 80 kg in study arm 4 (**S5 Fig**). The body weight-based dosing regimen was designed to keep median TEAEs probabilities below a 50% threshold. Simulated drug exposures, corresponding probabilities of adverse events (based on the logistic regression model) and total time over the target concentration are presented in **Fig 5**. Separate analyses for eye and nervous system TEAEs can be found in the Supplementary Information (**S6 Fig**). These results confirmed that twice daily dosing for 10 days is required in order to achieve target exposures in all body weight groups, while keeping TEAE probabilities below 50%.

## Discussion

MDA with ivermectin is one of the most successful public health interventions ever launched [29], with tremendous achievements made in reducing the morbidity and transmission of onchocerciasis in the African and American region [30]. However, the shift in paradigm from control to elimination poses major challenges, in particular in areas where conflict, civil unrest, epidemics, lack of political will or Loa-Loa co-endemicity hamper the achievement of regional and temporal treatment coverages that are sufficient to stop transmission. Moreover, to take the final steps towards onchocerciasis elimination, complete elimination mapping is essential in previously untreated or hypoendemic (often remote) areas where onchocerciasis was not considered to constitute a major public health problem [14].

Emodepside is a drug candidate with the potential to overcome some of these challenges. The macrofilaricidal compound could potentially be applied as a relatively short-course treatment for individual case management in order to support onchocerciasis elimination goals [31]. In this study, we investigated the population pharmacokinetic properties and exposure–adverse events relationships of emodespide after oral administration of an LSF solution or two immediate-release tablet formulations in healthy male subjects. Three phase I clinical trials, including a SAD, MAD and RelBA study, were pooled for the population pharmacokinetic analysis. The SAD study is a First-in-Human study and this is the first population pharmacokinetic analysis of emodepside in humans.

### Pharmacokinetic properties of emodepside

Emodepside plasma and DBS concentrations were well described by a transit-compartment absorption model, followed by a three-compartment disposition model. A transit absorption model yielded a better model fit as compared to a lag time model and is also a more physiological representation for the observed delay in emodepside absorption along the gastrointestinal tract [32].

Absorption was relatively rapid after administration of emodepside as an oral LSF solution in the fasted state (T_max_ = 1.22 h), while administration as a tablet formulation and food intake reduced the rate and also the extent of absorption. The data can be explained by incomplete and prolonged dissolution of emodepside in gastrointestinal fluids (tablet formulation) as well as slowed down gastric emptying (by food intake) [33]. Apart from formulation and food effects, dose had a significant impact on the rate of drug absorption. The investigated dose range (1–40 mg, corresponding to 0.012–0.667 mg emodepside per kg body weight per day) corresponds to a 70% maximum difference in MTT between the lowest and the highest dose. Emodepside exposure was dose-proportional in the present study population. However, as emodepside is a P-glycoprotein substrate [34], absorption-related saturation at higher, but clinically non relevant, doses cannot be excluded.

The three-compartment disposition model indicates a complex disposition behavior. Emodepside is highly lipophilic (logP = 4.9, Bayer, in-house data) and our results showed a moderate apparent volume of distribution for the central and the shallow peripheral compartment (V_c_/F = 52.4 L, V_p1_/F = 44.4 L), and a high apparent volume of distribution for the deep peripheral compartment (V_p2_/F = 647 L), pointing to extensive tissue distribution. The results are in line with biodistribution studies with ^14^C-labeled emodepside in rats, which revealed a moderate to high affinity to most tissues and organs. This is also consistent with the observed long terminal elimination half-life in this study (18.4 days) as a result of large drug distribution and low clearance (estimated as 1.29 L/h (95% CI 1.18–1.40 L/h)). The results of this study are in agreement with the previously published non-compartmental analysis of emodespide pharmacokinetics in healthy volunteers [27]. Fast absorption, long terminal half-life and dose-proportional increases in plasma concentration for emodepside doses up to 40 mg (for the LSF solution) have been reported. Moreover the same trends in terms of food and formulation effects on rate and extent of absorption were highlighted [27]. However, a major strength of this study is the use of nonlinear mixed-effects modeling in order to characterize the effects of different covariates with higher statistical power as compared to non-compartmental analysis. Apart from formulation, food and dose effects, body weight was identified as a significant covariate and was included in the population pharmacokinetic model by allometric scaling of all clearance and volume of distribution parameters. We used theory-based allometric exponents in the present study which have a strong biological foundation [35]. The absence of additional covariate effects for the present study population (healthy male, White men) does not exclude significant and clinically relevant covariate effects in a patient population with potentially larger heterogeneity in demographic and pharmacokinetic parameters. Further investigation of covariate effects in onchocerciasis patients is required to fully characterise the effects of e.g. disease state, body physiology and level of metabolising enzymes on emodepside pharmacokinetics. Another strength of the study was the dense sampling, both in the absorption and in the elimination phase, on the first day and tenth day (MAD study) of dosing, which allowed for the implementation of a highly flexible transit-absorption model, including parameterization of IOV on absorption rate.

### The relationship between drug exposure and adverse events

Emodepside was considered safe in all investigated dose groups. However, a single dose of 40 mg emodepside, given as LSF solution, was not well tolerated and 20 mg was considered the maximum tolerated dose in the SAD study, according to the safety review group. The occurrence of drug-related TEAE of interest was successfully modeled using a binary logistic regression approach and individual exposure data as explanatory variables. Peak plasma concentration was identified as a better TEAE predictor as compared to daily or cumulative dose or total exposure, which is in agreement with the majority of drug-related TEAEs of interest occurring 1–3 h post-dose, mostly with a short delay after T_max_. There was a pronounced increase in the percentage of subjects reporting eye disorder and central nervous system disorder TEAEs with increasing plasma peak concentrations. The underlying mechanism driving the adverse events is not yet understood. However, it is worth noting that neurotoxicity of emodepside was also observed in P-glycoprotein deficient mice and dogs, which was related to increased brain penetration of emodepside [34,36]. This potential interaction between P-glycoprotein activity and adverse events should be considered carefully for patient populations with co-morbidities receiving concomitant medications. Oxidative metabolism of emodepside has been shown to be predominantly catalyzed by CYP3A4. Therefore, drug-drug interactions (DDI) through interaction with P-glycoprotein as well as CYP3A4 cannot be excluded.

### Dose finding studies

The design of the proof-of concept phase II study was optimized to maintain total plasma concentrations in humans above C_target_ (100 ng/mL), a concentration showing antiparasitic activity *in vivo* in the *O*.*ochengi* cattle model [22]. An additional criterion for dosing selection was the duration of drug exposure above the target concentration, with a minimum of 5 days’ exposure assumed to be essential for macrofilaricidal efficiency as shown in an *L*.*sigmodontis*-infected rat model [28]. Pre-clinical models have been established as surrogate models of human filarial disease and advantages as well as limitations have been discussed previously [37]. Despite their value in drug discovery, pre-clinical nematode models remain an imperfect description of *O*.*volvulus* infections in human—not just in terms of the nature of the parasite and host but also regarding disease pathology, site of infection, presence/absence of nodules, etc. [37]. In terms of plasma protein binding, species differences were found to be relatively small between mice, dogs and humans (f_u,plasma_ = 1.0–1.6%). In rats, gerbils and rabbits, the fraction of unbound drug in plasma was slightly higher (f_u,plasma_ = 2.7–3.1% [38]). Taking into consideration that the total plasma concentration above C_target_ was associated with anthelmintic efficacy *in vivo*, these small differences in protein binding are unlikely to be clinically relevant. However, a limitation of our study is the uncertainty around the target concentration as well as the applicability of the pre-clinical efficacy studies to human. Prospective trials to assess the clinical efficacy of emodepside in onchocerciasis patients are urgently needed.

For the dose finding studies, the risk of under-dosing had to be balanced with the risk of the occurrence of drug-related TEAEs of interest. Since all drug-related TEAEs in the study population were transient and mild or moderate in severity, and no serious drug-related TEAEs were recorded, an acceptable tolerability was defined as < 50% probability for any drug-related TEAE of interest. The proposed body weight-based dosing regimen (twice daily dosing for 10 days, with ASD-tablet B, in fasted state) achieved prolonged emodepside exposures above C_target_ and acceptable tolerability for a wide range of body weights. A loading dose of emodepside (e.g. 20 mg, three times a day, on the first day of dosing) was evaluated also for faster achievement of C_target_ levels and a shorter treatment duration. However, as this resulted in C_max_ levels above the tolerability margins, a loading dose was not considered further.

## Conclusions

In summary, the present study reports the first population pharmacokinetic analysis of emodepside in humans, based on a pooled analysis of three phase I clinical trials, and using a nonlinear mixed-effects approach. The developed population pharmacokinetic model adequately described emodepside pharmacokinetics in healthy male subjects after administration of either an oral solution or two immediate-release tablet formulations. Formulation and food had a significant impact on relative bioavailability and absorption rate. Additional covariates, i.e. body weight and dose, were identified as influential to emodepside disposition and absorption rate, respectively.

Individual exposure–adverse events modeling and simulation was used to derive an optimized, body weight-based dosing regimen, which allowed for achievement of extended emodepside exposures above target concentrations while maintaining acceptable tolerability margins. A body weight-based dosing regimen is recommended, but implementation in the field might not be achievable and requires further evaluation. The proposed dosing regimen with 10 days of dosing (twice daily) would be a major step towards reaching onchocerciasis elimination goals compared to decade long MDA with ivermectin. Clinical efficiency of emodepside remains to be shown, and results from the present study support to move forward to clinical phase II as a next step on the road towards onchocerciasis elimination.

## Supporting information

S1 Table Summary of HPLC-MS/MS conditions. (DOCX)

S2 Table Key steps in the model building history for the population pharmacokinetic analysis of emodepside in NONMEM. (DOCX)

S3 Table Simulated secondary pharmacokinetic parameter estimates. Simulations are based on the final population pharmacokinetic model for emodepside and a proposed dosing regimen (10 mg emodepside, twice daily, for 10 days, for a 75 kg subject).
(DOCX)

S4 Table Drug-related treatment-emergent adverse events (TEAE). (DOCX)

S5 Table Summary of temporal characteristics of drug-related TEAE of interest. TEAEs of interest for the SAD, MAD and RelBA study are listed (only cohorts in which drug-related TEAEs of interest occurred).
(DOCX)

S6 Table Binary logistic regression model diagnostics. Model diagnostics shown for the correlation between exposure and the odds of experiencing a drug-related TEAEs of interest (eye disorder and/or nervous system disorder).
(DOCX)

S7 Table Binary logistic regression model diagnostics. Model diagnostics shown for the correlation between exposure and the odds of experiencing any drug-related TEAEs.
(DOCX)

S1 Fig Linear regression analysis (emodepside DBS vs. plasma concentrations). Observations are represented by black open circles, the black solid line represents the line of identity, and the black dotted line represents the linear regression. DBS concentration = 0.605 × plasma concentration-0.592 (r^2^ = 0.987, standard error of estimate, SEE = 5.74, n = 228).
(TIF)

S2 Fig Goodness-of-fit of the final population pharmacokinetic model for emodepside. **a:** observed versus population predicted concentrations. **b:** observed versus individually predicted concentrations. **c:** conditionally weighted residuals versus population predicted concentrations. **d:** conditionally weighted residuals versus time after dose. Observations are represented by grey circles, solid grey lines represent the line of identity or zero line, and the local polynomial regression fitting for all observations is represented by the dashed black line.
(TIF)

S3 Fig Receiver Operating Characteristics (ROC) curves for binary exposure–adverse event models. **a:** all drug-related TEAE of interest (eye disorder or nervous system disorder, whatever occurs first), **b:** eye disorder only, **c:** nervous system disorder only.
(TIF)

S4 Fig Simulations of pharmacokinetic profiles for emodepside for different dosing regimens. Simulations are based on the final population pharmacokinetic model, and the proposed route of administration for a planned Phase II clinical trial (ASD-tablet formulation B, fasted state, for a 75 kg adult). **a:** 10 mg, once daily, for 7 days, **b:** 10 mg, once daily, for 14 days, **c:** 10 mg, twice daily, for 7 days, and **d:** 10 mg, twice daily, for 10 days. Black solid lines represent the median of the simulated emodepside plasma concentrations over time, with the 90% prediction interval shown as shaded area (5^th^ and 95^th^ percentiles). The horizontal red line represents the target concentration (C_target_ = 100 ng/mL). Time between vertical dotted lines illustrate the duration of ***continuou***s mean drug concentration above C_target_ (with C_trough_ > C_target_ for 50% of the simulated population), i.e. 0 days (Scenario a, b); 2.5 days (Scenario c); 13 days (Scenario d). The corresponding ***total*** time above C_target_ (including intermittent time intervals were C_trough_ < C_target_) is 0.9 (0.0–2.0) days (Scenario a), 3.6 (1.0–16.5) days (Scenario b), 5.4 (1.6–17.2) days (Scenario c), and 15.9 (3.9–30.1) days (Scenario d). Values for total time above C_target_ are given as median (5^th^ to 95^th^ percentile).
(TIF)

S5 Fig Emodepside exposure and pedicted probabilities of drug-related TEAE of interest for fixed dosing with emodepside. Maximum emodepside plasma exposure (C_max_, upper panel), corresponding predicted probability of drug-related TEAE of interest (middle panel) and total time above the target concentration (100 ng/mL, lower panel), as a function of body weight. The midline of the boxplots indicates the median, the box corresponds to the interquartile range, and the whiskers extend from the 5th to the 95th percentile. The red dotted line in the middle panel indicates a 50% probability threshold for any drug-related TEAE of interest. The red dotted line in the lower panel indicates the minimum number of days (5 days) above the target concentration. Simulations are based on fixed dosing with 20 mg (**a:** study arm 1) and 10 mg (**b-d:** study arm 2–4) emodepside, administered in fasted state, as amorphous solid dispersion (ASD)-tablet B. **a:** study arm 1 (single dose of emodespide), **b:** study arm 2 (once daily dosing for 7 days), c: study arm 3 (once daily dosing for 14 days), **d:** study arm 4 (twice daily dosing for 10 days).
(TIF)

S6 Fig Predicted probabilities of drug-related TEAE of interest. **a:** study arm 1 (single dose of emodespide), **b:** study arm 2 (once daily dosing for 7 days), **c:** study arm 3 (once daily dosing for 14 days) and **d:** study arm 4 (twice daily dosing for 10 days), administered in fasted state, as amorphous solid dispersion (ASD)-tablet B. Body weight-based dosing according to Fig 4 in the main text. The midline of the boxplots indicates the median, the box corresponds to the interquartile range, and the whiskers extend from the 5th to the 95th percentile. The red dotted line indicates a 50% probability threshold for any drug-related TEAE of interest. Probabilities of drug-related TEAE were predicted for all TEAE of interest (either eye disorder or nervous system disorder, left panel). Stratified analysis for eye disorders (middle panel) and nervous system disorders (right panel) are shown separately.
(TIF)

S1 Text Rational for only including drug-related TEAEs in the logistic regression model for subsequent dose finding studies. (DOCX)

S1 Code NONMEM code for the final population pharmacokinetic model. (DOCX)
