# Supplementary material for: Drug development for the treatment of onchocerciasis: Population pharmacokinetic and adverse events modeling of emodepside
Source: PLoS Negl Trop Dis. 2022 Mar 10;16(3):e0010219. doi: 10.1371/journal.pntd.0010219 (PMC8912909; doi:10.1371/journal.pntd.0010219)
Supplement: S1 Table — (DOCX) [file pntd.0010219.s001.docx]

**S1 Table.** Summary of HPLC-MS/MS conditions

|  | **Human plasma** | **Dry blood spots** |
| --- | --- | --- |
| **Calibration range** | 1 – 800 ng/mL | 1 – 1000 ng/mL |
| **Injection volume** | 5 µL | 10 µL |
| **HPLC pump** | 1100 series (Agilent Technologies Inc, Santa Clara, CA, USA) | 1200 series pump (Agilent Technologies Inc, Santa Clara, CA, USA) |
| **Autosampler** | 1100 series (Agilent Technologies Inc, Santa Clara, CA, USA) | PAL (CTC Analytics AG, Zwingen, Switzerland) |
| **Column oven** | 1100 series (Agilent Technologies Inc, Santa Clara, CA, USA) | HotDog 5090 (Prolab GmbH, Reinach, Switzerland) |
| **Mobile phase** | Isocratic: Methanol (91% v/v), Water (9% v/v) with ammonium formate (0.5% m/v) | Phase A: water containing 2 mM ammonium acetate  Phase B: acetonitrile |
| **LC column** | Alltima^TM^ C18 5µM 150mm x 2.1 mm  (Grace) | Reproshell C18, 2 x 30 mm, 2.6 μm (Dr. Maisch HPLC GmbH, Ammerbuch, Germany) |
| **Mass spectrometer**  **(MS/MS)** | API4000^TM^ Turbo  (Applied Biosystems)  Ion source: electrospray ionization in positive ion mode  scan type: MRM  Mass (amu): 1119.5 (parent), 343.20 (product ion) | TSQ Vantage (Thermo Fisher Scientific, San Jose, CA, USA)  Ion source: electrospray ionization in positive ion mode  Scan type: SRM  Mass (amu): 1119.659 (parent), 343.200 (product ion) |
| **Inter-batch precision (% CV)** | 1.22% to 5.31 % | 3.2 % to 9.3 % |
| **Inter-batch accuracy (%)** | 99.0% to 101.4% | 100.8 % to 110.0 % |
