# Supplementary material for: Drug development for the treatment of onchocerciasis: Population pharmacokinetic and adverse events modeling of emodepside
Source: PLoS Negl Trop Dis. 2022 Mar 10;16(3):e0010219. doi: 10.1371/journal.pntd.0010219 (PMC8912909; doi:10.1371/journal.pntd.0010219)
Supplement: S2 Table — (DOCX) [file pntd.0010219.s002.docx]

# S2 Table. Key steps in the model building history for the population pharmacokinetic analysis of emodespide in NONMEM.

| **Model** | **Description** | **OFV** | **∆OFV** | **Comment** |
| --- | --- | --- | --- | --- |
| 1a | 1 comp, 1^st^ order absorption | 2318.4 | - |  |
| 1b | 2 comp, 1^st^ order absorption | -2642.8 | -4961.2 (reference: 1a) |  |
| 1c | **3 comp**, 1^st^ order absorption | -2808.0 | -165.1 (reference: 1b) |  |
| 1d | 4 comp, 1^st^ order absorption | -2954.8 | -165.1 (reference: 1c) | low precision^a^ |
| 2 | 3 comp, 1^st^ order absorption, **rel. bioav (F1)** | -2998.1 | -190.2 (reference: 1c) |  |
| 3a | 3 comp, 1^st^ order absorption, rel. bioav (F1), **lag time** | -6267.8 | -3269.5 (reference: 2) |  |
| 3b_1 | 3 comp, 1^st^ order absorption, rel. bioav (F1), 1 transit comp | -4715.3 | -1717.0 (reference: 2) |  |
| 3b_2 | 3 comp, 1^st^ order absorption, rel. bioav (F1), 2 transit comp | -5821.6 | -2823.4 (reference: 2) |  |
| 3b_3 | 3 comp, 1^st^ order absorption, rel. bioav (F1), 3 transit comp | -6386.1 | -3387.8 (reference: 2) |  |
| 3b_4 | 3 comp, 1^st^ order absorption, rel. bioav (F1), **4 transit comp** | -6589.4 | -3591.2 (reference: 2)  -321.6 (reference: 3a) |  |
| 3b_5 | 3 comp, 1^st^ order absorption, rel. bioav (F1), 5 transit comp | -6445.8 | -3447.6 (reference: 2) |  |
| 4 | 3 comp, 1^st^ order absorption, rel. bioav (F1), 4 transit comp,  **allometric scaling** | -6624.4 | -35.0 (reference: 3b_4) |  |
| 5 | 3 comp, 1^st^ order absorption, rel. bioav (F1), 4 transit comp,  allometric scaling, **Formulation on MTT** | -6757.0 | -132.7 (reference: 4) |  |
| 6 | 3 comp, 1^st^ order absorption, rel. bioav (F1), 4 transit comp,  allometric scaling, **Formulation on** MTT and **F1** | -6829.9 | -72.9 (reference: 5) |  |
| 7 | 3 comp, 1^st^ order absorption, rel. bioav (F1), 4 transit comp,  allometric scaling, Formulation on MTT and F1, **Food on MTT** | -6886.6 | -56.6 (reference: 6) |  |
| 8 | 3 comp, 1^st^ order absorption, rel. bioav (F1), 4 transit comp,  allometric scaling, Formulation on MTT and F1, **Food on** MTT and **F1** | -6917.3 | -30.8 (reference: 7) |  |
| 9 | 3 comp, 1^st^ order absorption, rel. bioav (F1), 4 transit comp,  allometric scaling, Formulation on MTT and F1, Food on MTT and F1, **IOV on MTT** | -6997.9 | -80.5 (reference: 8) |  |
| 10 | 3 comp, 1^st^ order absorption, rel. bioav (F1), 4 transit comp,  allometric scaling, Formulation on MTT and F1, Food on MTT and F1, **no IIV for SF** | -6986.3 | 11.6 (reference: 9) | Insufficient data to support IIV^b^ |
| 11 | 3 comp, 1^st^ order absorption, rel. bioav (F1), 4 transit comp,  allometric scaling, Formulation on MTT and F1, Food on MTT and F1, **no IIV for SF**, **Dose on MTT** | -6997.4 | -11.17 (reference: 10) | Final model |
|  |  |  |  |  |

In grey are presented models that were moved forward. ^a^ relative standard errors, RSE, of 37-1192 %, ^b^Dry blood spot (DBS) data only available from one cohort. **Abbreviations:** comp, compartment; rel.bioav (F1), relative bioavailability; MTT, mean transit time; IOV, interoccasion variability; IIV interindividual variability; SF, venous plasma – DBS scaling factor
