# Supplementary material for: Drug development for the treatment of onchocerciasis: Population pharmacokinetic and adverse events modeling of emodepside
Source: PLoS Negl Trop Dis. 2022 Mar 10;16(3):e0010219. doi: 10.1371/journal.pntd.0010219 (PMC8912909; doi:10.1371/journal.pntd.0010219)
Supplement: S3 Table — Simulations are based on the final population pharmacokinetic model for emodepside and a proposed dosing regimen (10 mg emodepside, twice daily, for 10 days, for a 75 kg subject). (DOCX) [file pntd.0010219.s003.docx]

**S3 Table.** Simulated secondary pharmacokinetic parameter estimates. Simulations are based on the final population pharmacokinetic model for emodepside and a proposed dosing regimen (10 mg emodepside, twice daily, for 10 days, for a 75 kg subject).

| **Parameter** | **Oral LSFsolution** | |  | **ASD-tablet A** | |  | **ASD-tablet B** | |
| --- | --- | --- | --- | --- | --- | --- | --- | --- |
|  | Fasted | Fed |  | Fasted | Fed |  | Fasted | Fed |
| C_max_ (ng/mL) | 348 (236 – 510) | 245 (169 – 360) |  | 211 (146 – 307) | 145 (99 – 212) |  | 258 (178 – 378) | 179 (123 – 262) |
| T_max_ (h) | 1.11 (0.57 – 2.17) | 2.09 (1.08 – 4.02) |  | 3.09 (1.60 – 5.85) | 5.67 (3.00 – 10.11) |  | 2.38 (1.23 – 4.54) | 4.40 (2.31 – 8.15) |
| AUC_∞_ (μg×h/mL) | 155 (96 – 246) | 117 (73 – 186) |  | 106 (66 – 169) | 80 (50 – 127) |  | 124 (77 – 197) | 94 (58 – 149) |
| t_½_ (days) | 18.4 (11.0 – 32.7) | |  | 18.4 (11.0 – 32.7) | |  | 18.4 (11.0 – 32.7) | |

All values are given as median (5^th^ to 95^th^ percentile). **Abbreviations:** ASD tablet, amorphous solid dispersion tablet; C_max_, maximum concentration for the last dose (10^th^ day of dosing); T_max_, time after dose to reach the maximum concentration (calculated as median (5^th^ to 95^th^ percentile) across all dosing events); AUC_∞_, area under the concentration-time curve from the first dose to infinity (~10 half-lives after the last dose); t_1/2_ terminal elimination half-life
