# Supplementary material for: Drug development for the treatment of onchocerciasis: Population pharmacokinetic and adverse events modeling of emodepside
Source: PLoS Negl Trop Dis. 2022 Mar 10;16(3):e0010219. doi: 10.1371/journal.pntd.0010219 (PMC8912909; doi:10.1371/journal.pntd.0010219)
Supplement: S4 Table — (DOCX) [file pntd.0010219.s004.docx]

**S4 Table.** Drug-related treatment-emergent adverse events (TEAE).

| Drug-related TEAE | Subjects experiencing TEAEs (n = 142) | % of subjects |
| --- | --- | --- |
| **Eye disorders^a^** | **20** | **14.1** |
| Visual impairment | 13 | 9.2 |
| Vision blurred | 7 | 4.9 |
| Photophobia | 2 | 1.4 |
| Eye pain | 1 | 0.7 |
| Photopsia | 1 | 0.7 |
| Visual acuity reduced | 1 | 0.7 |
| **Nervous system disorders^a^** | **18** | **12.7** |
| Dizziness | 7 | 4.9 |
| Headache | 6 | 4.2 |
| Feeling of relaxation | 3 | 2.1 |
| Euphoric mood | 2 | 1.4 |
| Balance disorder | 1 | 0.7 |
| Disorientation | 1 | 0.7 |
| Somnolence | 1 | 0.7 |
| **Gastrointestinal disorders** | **4** | **2.8** |
| Nausea | 3 | 2.1 |
| Abdominal pain | 1 | 0.7 |
| Paraesthesia oral | 1 | 0.7 |
| Vomiting | 1 | 0.7 |
| **Psychiatric disorders** | **2** | **1.4** |
| Disturbance in attention | 1 | 0.7 |
| Hypervigilance | 1 | 0.7 |
| Nervousness | 1 | 0.7 |
| **General disorders and administration site conditions** | **1** | **0.7** |
| Sensation of foreign body | 1 | 0.7 |
| **Musculoskeletal and connective tissue disorders** | **1** | **0.7** |
| Muscle spasms | 1 | 0.7 |
| **Respiratory, thoracic and mediastinal disorders** | 1 | 0.7 |
| Cough | 1 | 0.7 |
| Oropharyngeal pain | 1 | 0.7 |
| **Skin and subcutaneous tissue disorders** | 1 | 0.7 |
| Pigmentation disorder | 1 | 0.7 |

^a^ Eye disorder and nervous system disorder were identified as drug-related TEAE of interest.
