# Supplementary material for: Drug development for the treatment of onchocerciasis: Population pharmacokinetic and adverse events modeling of emodepside
Source: PLoS Negl Trop Dis. 2022 Mar 10;16(3):e0010219. doi: 10.1371/journal.pntd.0010219 (PMC8912909; doi:10.1371/journal.pntd.0010219)
Supplement: S5 Table — TEAEs of interest for the SAD, MAD and RelBA study are listed (only cohorts in which drug-related TEAEs of interest occurred). (DOCX) [file pntd.0010219.s005.docx]

**S5 Table.** Summary of temporal characteristics of drug-related TEAE of interest. TEAEs of interest for the SAD, MAD and RelBA study are listed (only cohorts in which drug-related TEAEs of interest occurred).

| **Study Arm** | **N^a^** | **TEAE by organ class** | **Severity of TEAE** | **Time of TEAE (h)^b^** | **Tmax (h)** | **Lag time (h) of TEAE relative to Tmax ^b^** | **Duration of TEAE (h) ^b^** |
| --- | --- | --- | --- | --- | --- | --- | --- |
| **SAD study** | | | | | | | |
| 1 mg LSF, fasted | 1/ 5 | Nervous system disorders | Mild | 1h | 57min | 2min | 3h20min |
| 5 mg LSF, fasted | 1/ 6 | Nervous system disorders | Mild | 2h | 1h04min | 55min | 29h31min |
| 10 mg LSF, fasted | 3/6 | Nervous system disorders | Mild | 4h50min | 1h04min | 3h45min | 16h30min |
|  |  | Eye disorders | Mild | 1h50min | 58min | 51min | 4h39min |
|  |  | Eye disorders | Mild | 2h15min | 59min | 1h15min | 3h34min |
| 20 mg LSF, fasted | 2/ 6 | Nervous system disorders | Mild | 5h30min | 1h36min | 3h53min | 54min |
|  |  | Eye disorders | Mild | 1h10min | 1h10min | at Tmax | 1h07min |
| 40 mg LSF, fasted (Cohort 8) | 5 / 6 | Eye disorders | Mild | 0h30min | 1h25min | before Tmax | 21h40min |
|  |  | Nervous system / eye disorders | Mild | 1h35min; 2h35min | 1h48min | before Tmax; 46min | 20h39min; 22h10min |
|  |  | Eye disorders | Mild | 1h50min | 1h20min | 29min | 19h14min |
|  |  | Nervous system / eye disorders | Mild | 3h02min; 3h30min | 50min | 2:11; 2:39 | 6h43min; 10h14min |
|  |  | Eye / nervous system disorders | Mild | 0h30min; 0h20min; 0h20min | 1h17min | before Tmax | 20h29min; 20h39min; 8h55min |
| 10 mg, LSF, fed | 2/6 | Nervous system disorders | Mild | 2h38min | 1h30min | 1h07min | 6h27min |
|  |  | Nervous system disorders | Mild | 2h22min | 1h26min | 55min | 3h47min |
| 40 mg LSF, fasted (Cohort 10) | 5/6 | Nervous system disorders | Moderate | 5h30min | 1h03min | 4h26min | 7h59min |
|  |  | Nervous system / eye disorders | Mild | 2h45min; 3h55min; 3h45min; 4h20min | 1h53min | 51min; 2h01min; 1h51min; 2h26min | 3h54min; 3h44min; 19h10min; 46h55min |
|  |  | Nervous system / eye disorders | Mild / moderate | 1h15min; 2h | 1h49min | before Tmax; 10min | 10h45min; 7h14min |
|  |  | Eye / nervous system disorders | Mild | 1h50min; 1h50min; 1h50min; 1h15min | 1h33min | 0:16; before Tmax | 7h35min; 7h35min; 7h35min; 8h09min |
|  |  | Eye / nervous system disorders | Mild | 2h50min; 1h00min; 2h45min; 1h15min | 1h06min | 1h43min; before Tmax; 1h38min; 8min | 1h; 20h49min; 34min; 20h35min |
| **MAD study** | | | | | | | |
| 5mg, solution, fasted, QD | 2 / 6 | Nervous system / eye disorders | Mild | 2h17min; 0h25min; 0h25min | 47min | 1h29min; before Tmax | 2h28min; 34min; 34min |
|  |  | Eye disorders | Mild | 1h; 1h | 49min | 10min; 10min | 1h59min; 1h59min |
| 10mg, solution, fasted, QD | 1 / 6 | Nervous system / eye disorders | Mild | 2h05min; 2h05min; 1h | 50min | 1h14min; 1h14min; 9min | 2h25min; 30min; 2h45min |
| 10mg, solution, fasted, BID | 3 / 6 | Eye disorders | Mild | 2h | 1h49min | 10min | 48h |
|  |  | Eye disorders | Mild | 1h45min | 1h13min | 31min | 48h |
|  |  | Eye disorders | Mild | 2h30min | 1h09min | 1h20min | 504h |
| **RelBA study** | | | | | | | |
| 5mg, Tablet # 416, fasted | 1/ 10 | Nervous system / eye disorders | Mild | 2h15min; 2h15min | 2h09min | 5min; 5min | 1h35min; 1h35min |
| 10mg, Tablet # 416, fasted | 1/10 | Nervous system / eye disorders | Mild | 1h25min; 1h25min | 2h07min | before Tmax | 4h45min |

^a^ number subjects with drug-related TEAE of interest / subjects exposed to emodepside. ^b^ several time points, lag times and durations (with number of listings > number of TEAEs by organ class) indicate that a subjects reported several specific drug-related TEAEs of interest (by preferred term), such as blurred vision and visual impairment (eye disorders) or headache and dizzinies (nervous system disorders). **Abbreviations:** single ascending dose, SAD; LSF, liquid service formulation
