# Supplementary material for: Drug development for the treatment of onchocerciasis: Population pharmacokinetic and adverse events modeling of emodepside
Source: PLoS Negl Trop Dis. 2022 Mar 10;16(3):e0010219. doi: 10.1371/journal.pntd.0010219 (PMC8912909; doi:10.1371/journal.pntd.0010219)
Supplement: S6 Table — Model diagnostics shown for the correlation between exposure and the odds of experiencing a drug-related TEAEs of interest (eye disorder and/or nervous system disorder). (DOCX) [file pntd.0010219.s006.docx]

**S6 Table.** Binary logistic regression model diagnostics. Model diagnostics shown for the correlation between exposure and the odds of experiencing a drug-related TEAEs of interest (eye disorder and/or nervous system disorder).

| Parameter | C_max_ | | |  | AUC_∞_ | | |  | Daily Dose | | |  | Cumulative Dose | | |
| --- | --- | --- | --- | --- | --- | --- | --- | --- | --- | --- | --- | --- | --- | --- | --- |
|  | AE of interest | Eye disorder | Nervous system disorder |  | AE of interest | Eye disorder | Nervous system disorder |  | AE of interest | Eye disorder | Nervous system disorder |  | AE of interest | Eye disorder | Nervous system disorder |
| AIC | 107.1 | 82.1 | 90.0 |  | 133.7 | 107.2 | 111.4 |  | 106.7 | 84.7 | 90.3 |  | 84.7 | 107.3 | 111.6 |
| Log Likelihood | -51.56 | -39.0 | -43.0 |  | -64.8 | -51.6 | -53.7 |  | -51.4 | -40.4 | -43.1 |  | -64.7 | -51.6 | -53.8 |
| Mc Fadden R^2^ | 0.25^***^ | 0.32^***^ | 0.20^***^ |  | 0.06^**^ | 0.11^***^ | 0.01 |  | 0.26^***^ | 0.30^***^ | 0.20^***^ |  | 0.06^**^ | 0.11^***^ | 0.00 |
| **Accuracy** | **87.3** | **91.6** | **90.1** |  | **82.4** | **87.3** | **87.3** |  | **86.6** | **90.1** | **90.1** |  | **81.0** | **87.3** | **87.3** |
| **ROC, area (%)** | **83.2** | **89.7** | **77.6** |  | **79.9** | **85.5** | **72.7** |  | **79.9** | **83.8** | **75.1** |  | **79.1** | **85.4** | **71.3** |
| **Odds (%)**  **(95% CI)^a^** | **0.77**  **(0.47-1.08)** | **0.86**  **(0.53-1.19)** | **0.63**  **(0.35-0.91)** |  | **1.50**  **(0.48-2.54)** | **1.89**  **(0.81-2.99)** | **0.49**  **(-0.69-1.67)** |  | **12.8**  **(7.46-18.5)** | **12.9**  **(7.77-18.3)** | **9.85**  **(5.46-14.4)** |  | **1.26**  **(0.41-2.12)** | **1.55**  **(0.66-2.46)** | **0.34**  **(-0.65-1.34)** |
| LogOdds  (StError) | 0.0077^***^  (0.0015) | 0.0085^***^  (0.0017) | 0.0063^***^  (0.0014) |  | 0.0149^**^  (0.0052) | 0.0188^***^  (0.0055) | 0.0049  (0.0060) |  | 0.1207^***^  (0.0249) | 0.1215^***^  (0.0238) | 0.0939^***^  (0.0208) |  | 0.0126^**^  (0.0043) | 0.0154^***^  (0.0045) | 0.0034  (0.0051) |
| Intercept  (StError) | -2.87^***^  (0.40) | -3.59^***^  (0.51) | -3.20^***^  (0.45) |  | -1.79^***^  (0.26) | -2.30^***^  (0.31) | -2.03^***^  (0.29) |  | -2.97^***^  (0.41) | -3.52^***^  (0.48) | -3.24^***^  (0.45) |  | -1.82^***^  (0.26) | -2.32^***^  (0.31) | -2.02^***^  (0.29) |

**Abbreviations:** C_max_ maximum plasma emodepside concentration; AIC, Aikaike information criterion; n.d.; not defined, confusion matrix contains one group with 0 observations (division by 0), therefore sensitivity and specificity cannot be calculated (division by 0); ROC, area, area under the Receiver Operating Characteristics (ROC) curve.

^a^increase in odds of a drug-related TEAE per unit increase in C_max_ (ng/mL), AUC_∞_ (ug×h/mL), dose per day (mg) and cumulative dose (mg). ^***^ p value < 0.001, ^**^ p value < 0.01
