# Supplementary material for: Drug development for the treatment of onchocerciasis: Population pharmacokinetic and adverse events modeling of emodepside
Source: PLoS Negl Trop Dis. 2022 Mar 10;16(3):e0010219. doi: 10.1371/journal.pntd.0010219 (PMC8912909; doi:10.1371/journal.pntd.0010219)
Supplement: S7 Table — Model diagnostics shown for the correlation between exposure and the odds of experiencing any drug-related TEAEs. (DOCX) [file pntd.0010219.s007.docx]

**S7 Table.** Binary logistic regression model diagnostics. Model diagnostics shown for the correlation between exposure and the odds of experiencing any drug-related TEAEs.

| Parameter | C_max_ | AUC_∞_ | Daily Dose |  | Cumulative Dose |
| --- | --- | --- | --- | --- | --- |
| AIC | 125.8 | 146.7 | 125.0 |  | 146.5 |
| Log Likelihood | -60.9 | -71.4 | -60.5 |  | -71.2 |
| Mc Fadden R^2^ | 0.18 | 0.04 | 0.19 |  | 0.04 |
| **Accuracy** | **84.5** | **79.6** | **83.8** |  | **78.2** |
| **ROC, area (%)** | **75.2** | **72.6** | **72.7** |  | **71.2** |
| **Odds (%)**  **(95% CI)^a^** | **0.64**  **(0.37-0.92)** | **1.27**  **(0.27-2.27)** | **10.8**  **(5.99-15.8)** |  | **1.07**  **(0.24-1.90)** |
| LogOdds  (StError) | 0.0064^***^  (0.0014) | 0.0127*  (0.0050) | 0.1023  (0.0225) |  | 0.0106  (0.0042) |
| Intercept  (StError) | -2.38  (0.34) | -1.55  (0.24) | -2.51  (0.36) |  | -1.57  (0.24) |

**Abbreviations:** C_max_ maximum plasma emodepside concentration; AIC, Aikaike information criterion; ROC, area, area under the Receiver Operating Characteristics (ROC) curve.

^a^increase in odds of a drug-related TEAE per unit increase in C_max_ (ng/mL), AUC_∞_ (ug×h/mL), dose per day (mg) and cumulative dose (mg). ^***^ p value < 0.001, ^**^ p value < 0.01
