# Supplementary figures and images for: Drug development for the treatment of onchocerciasis: Population pharmacokinetic and adverse events modeling of emodepside
Source: PLoS Negl Trop Dis. 2022 Mar 10;16(3):e0010219. doi: 10.1371/journal.pntd.0010219 (PMC8912909; doi:10.1371/journal.pntd.0010219)

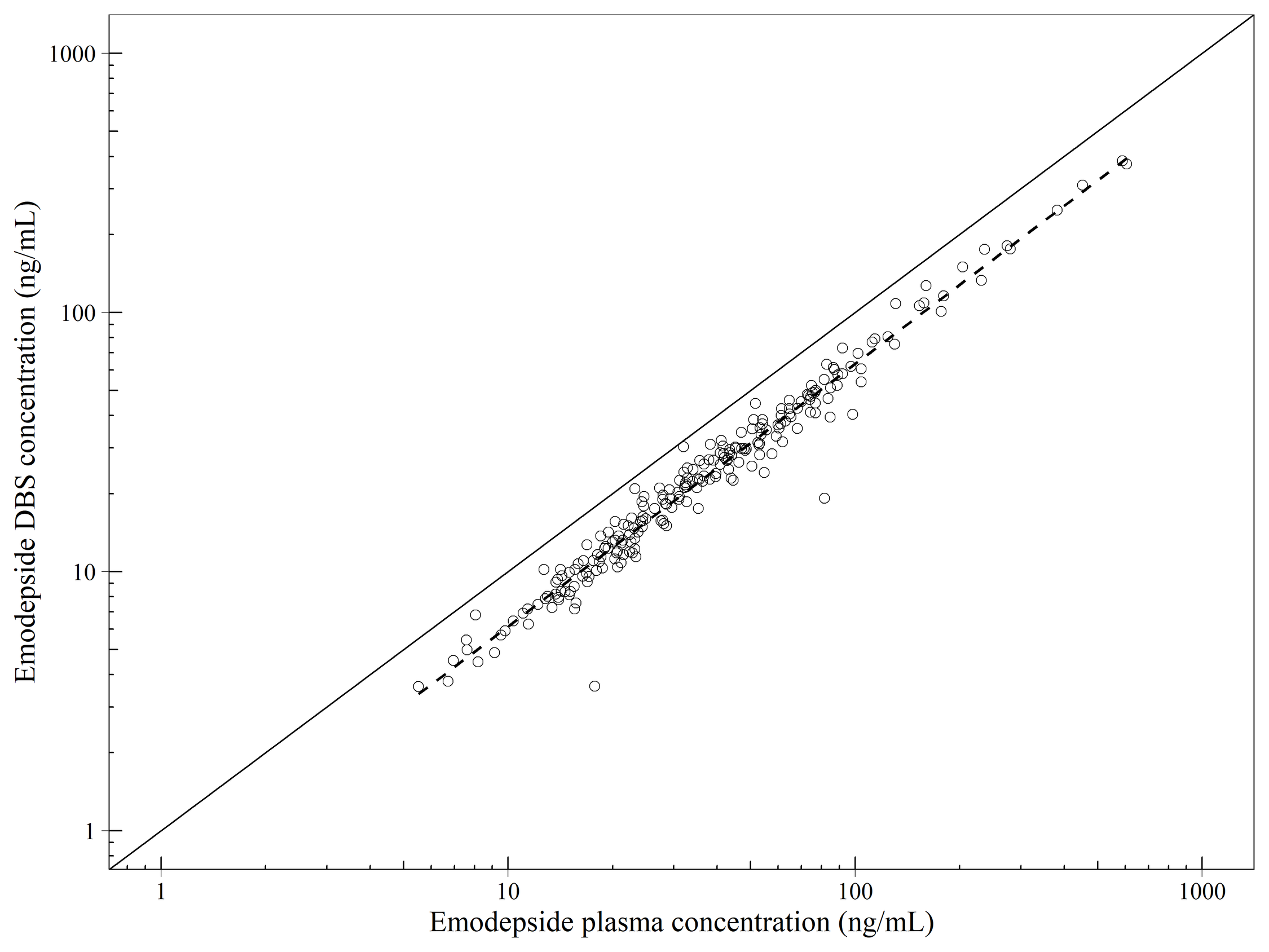

Supplement: S1 Fig — Observations are represented by black open circles, the black solid line represents the line of identity, and the black dotted line represents the linear regression. DBS concentration = 0.605 × plasma concentration-0.592 (r2 = 0.987, standard error of estimate, SEE = 5.74, n = 228). (TIF) [file pntd.0010219.s008.tif]

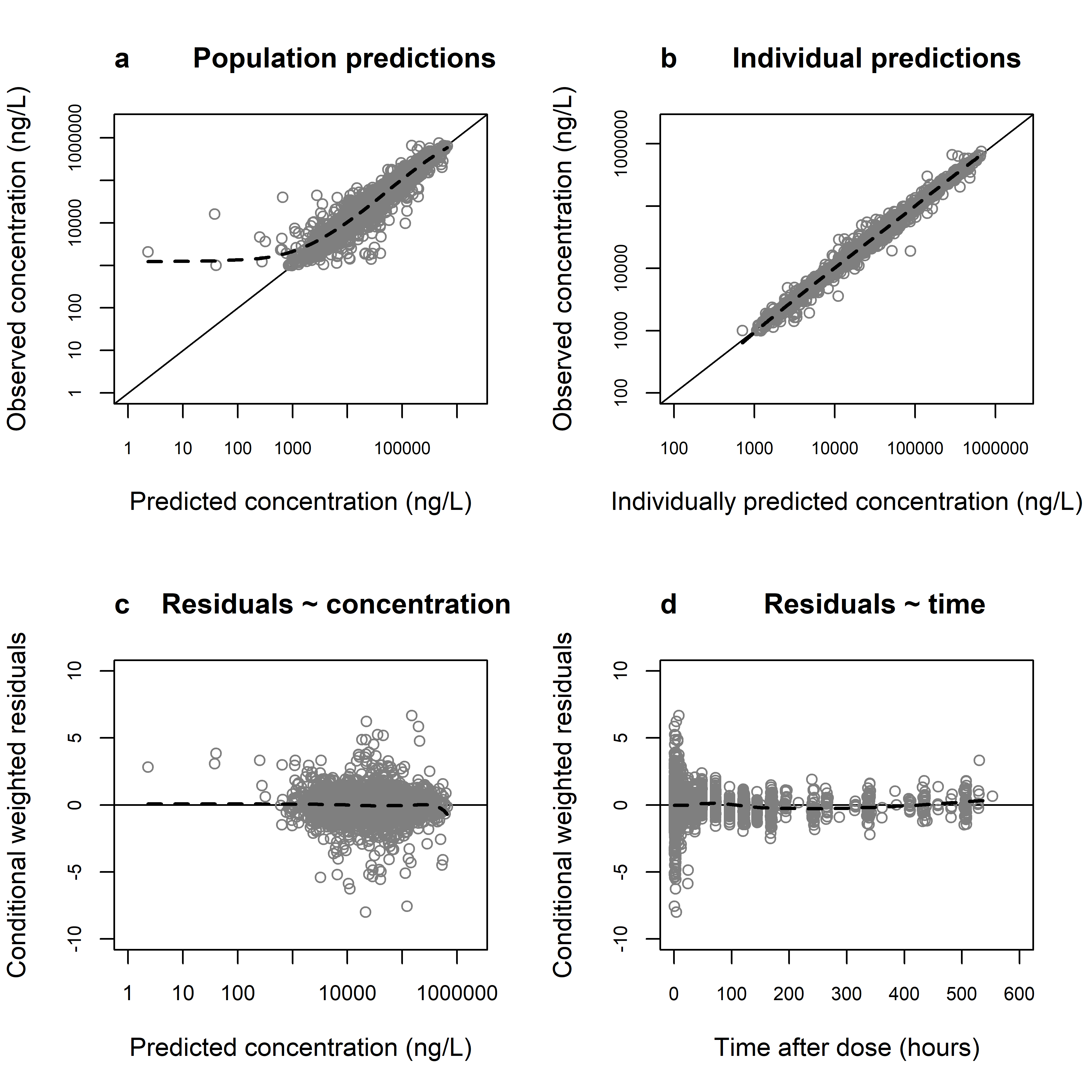

Supplement: S2 Fig — a: observed versus population predicted concentrations. b: observed versus individually predicted concentrations. c: conditionally weighted residuals versus population predicted concentrations. d: conditionally weighted residuals versus time after dose. Observations are represented by grey circles, solid grey lines represent the line of identity or zero line, and the local polynomial regression fitting for all observations is represented by the dashed black line. (TIF) [file pntd.0010219.s009.tif]

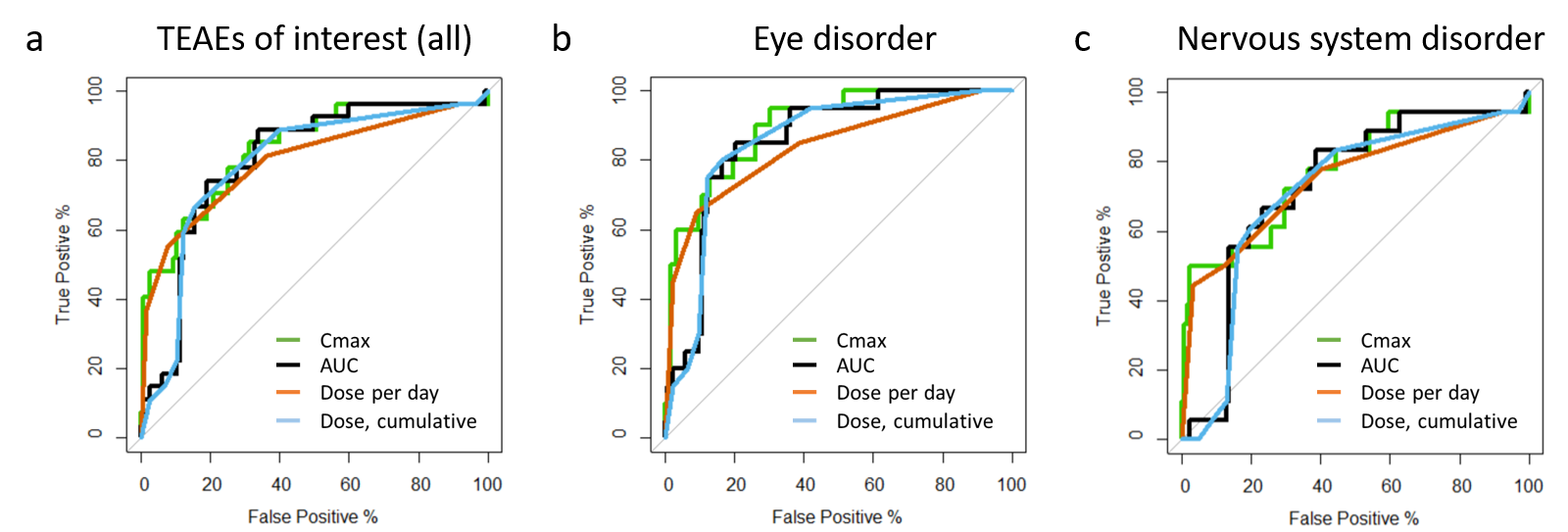

Supplement: S3 Fig — a: all drug-related TEAE of interest (eye disorder or nervous system disorder, whatever occurs first), b: eye disorder only, c: nervous system disorder only. (TIF) [file pntd.0010219.s010.tif]

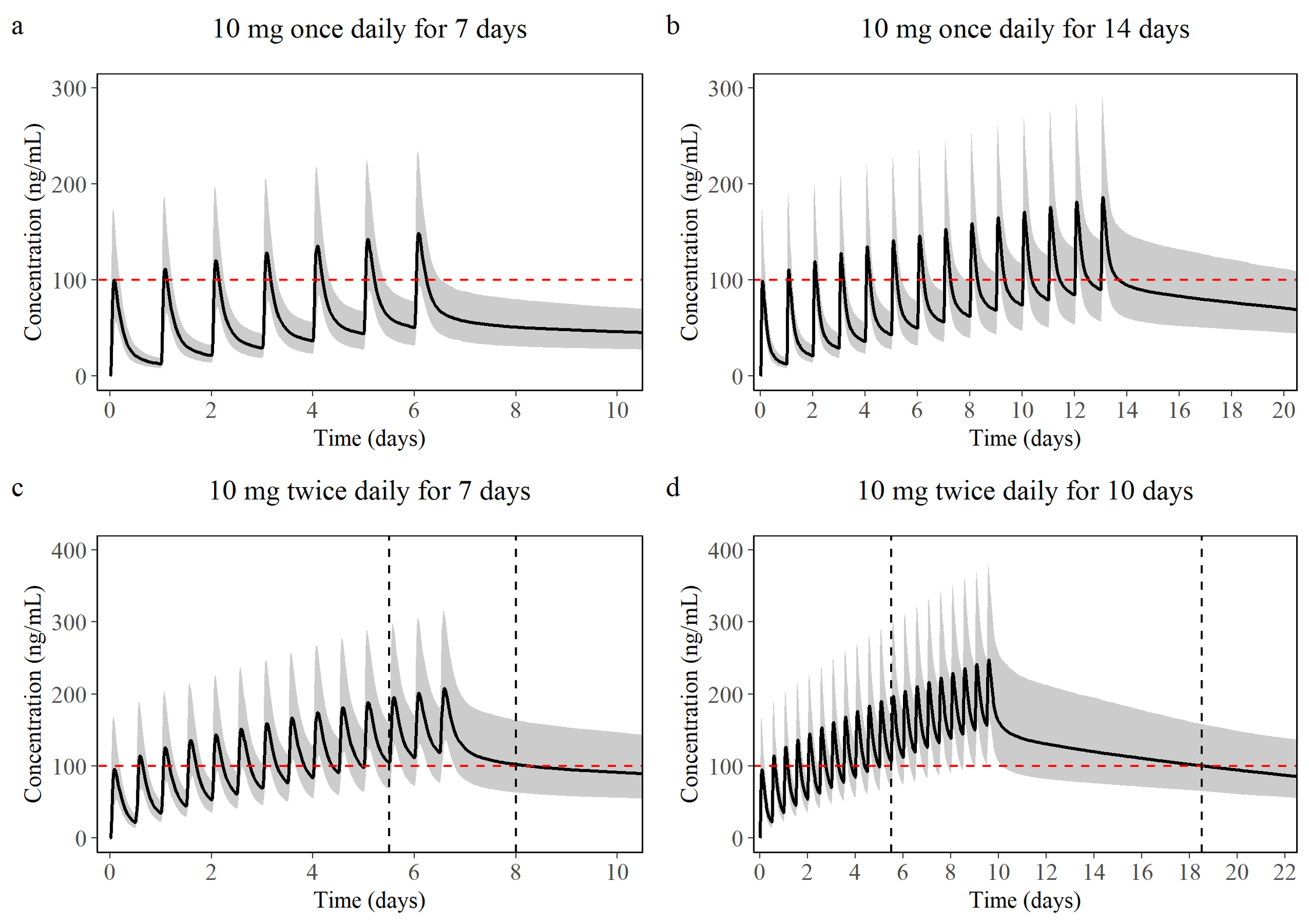

Supplement: S4 Fig — Simulations are based on the final population pharmacokinetic model, and the proposed route of administration for a planned Phase II clinical trial (ASD-tablet formulation B, fasted state, for a 75 kg adult). a: 10 mg, once daily, for 7 days, b: 10 mg, once daily, for 14 days, c: 10 mg, twice daily, for 7 days, and d: 10 mg, twice daily, for 10 days. Black solid lines represent the median of the simulated emodepside plasma concentrations over time, with the 90% prediction interval shown as shaded area (5th and 95th percentiles). The horizontal red line represents the target concentration (Ctarget = 100 ng/mL). Time between vertical dotted lines illustrate the duration of continuous mean drug concentration above Ctarget (with Ctrough > Ctarget for 50% of the simulated population), i.e. 0 days (Scenario a, b); 2.5 days (Scenario c); 13 days (Scenario d). The corresponding total time above Ctarget (including intermittent time intervals were Ctrough < Ctarget) is 0.9 (0.0–2.0) days (Scenario a), 3.6 (1.0–16.5) days (Scenario b), 5.4 (1.6–17.2) days (Scenario c), and 15.9 (3.9–30.1) days (Scenario d). Values for total time above Ctarget are given as median (5th to 95th percentile). (TIF) [file pntd.0010219.s011.tif]

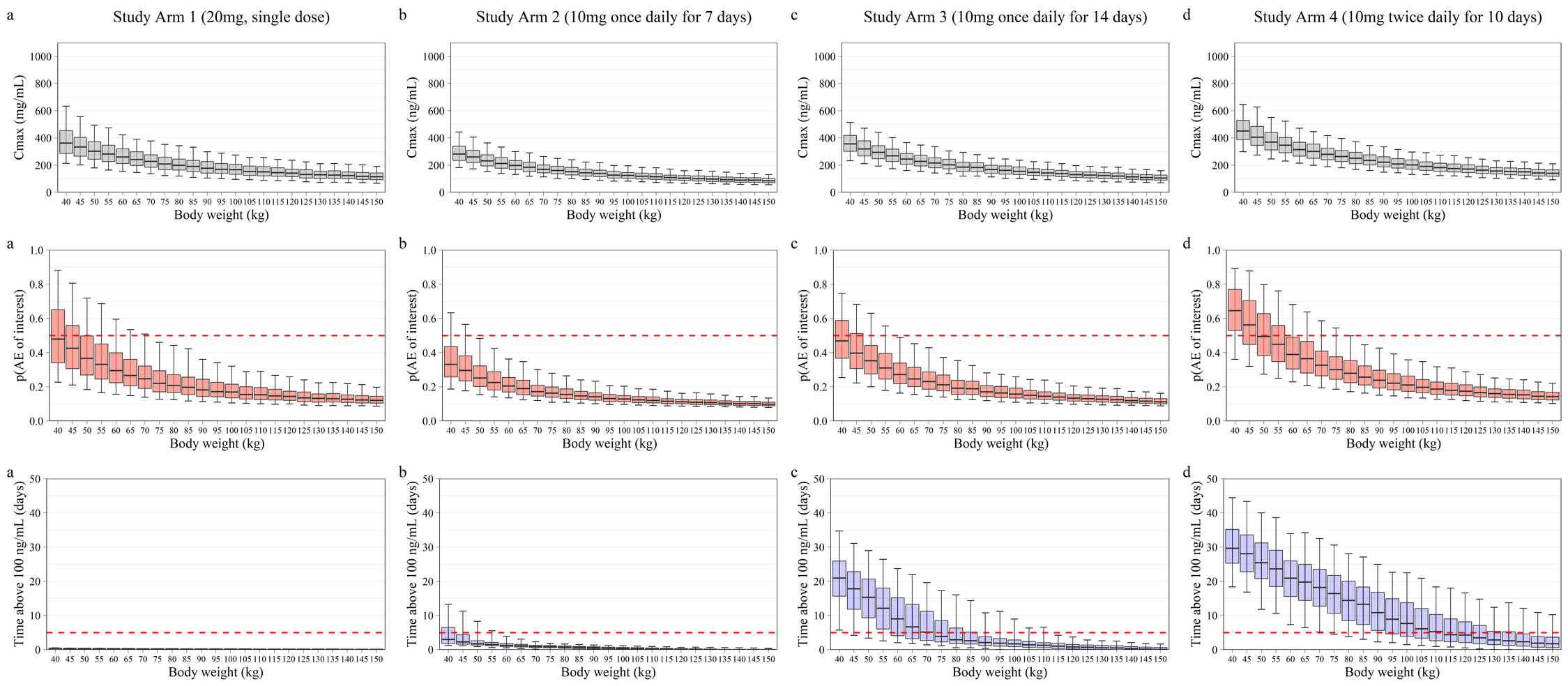

Supplement: S5 Fig — Maximum emodepside plasma exposure (Cmax, upper panel), corresponding predicted probability of drug-related TEAE of interest (middle panel) and total time above the target concentration (100 ng/mL, lower panel), as a function of body weight. The midline of the boxplots indicates the median, the box corresponds to the interquartile range, and the whiskers extend from the 5th to the 95th percentile. The red dotted line in the middle panel indicates a 50% probability threshold for any drug-related TEAE of interest. The red dotted line in the lower panel indicates the minimum number of days (5 days) above the target concentration. Simulations are based on fixed dosing with 20 mg (a: study arm 1) and 10 mg (b-d: study arm 2–4) emodepside, administered in fasted state, as amorphous solid dispersion (ASD)-tablet B. a: study arm 1 (single dose of emodespide), b: study arm 2 (once daily dosing for 7 days), c: study arm 3 (once daily dosing for 14 days), d: study arm 4 (twice daily dosing for 10 days). (TIF) [file pntd.0010219.s012.tif]

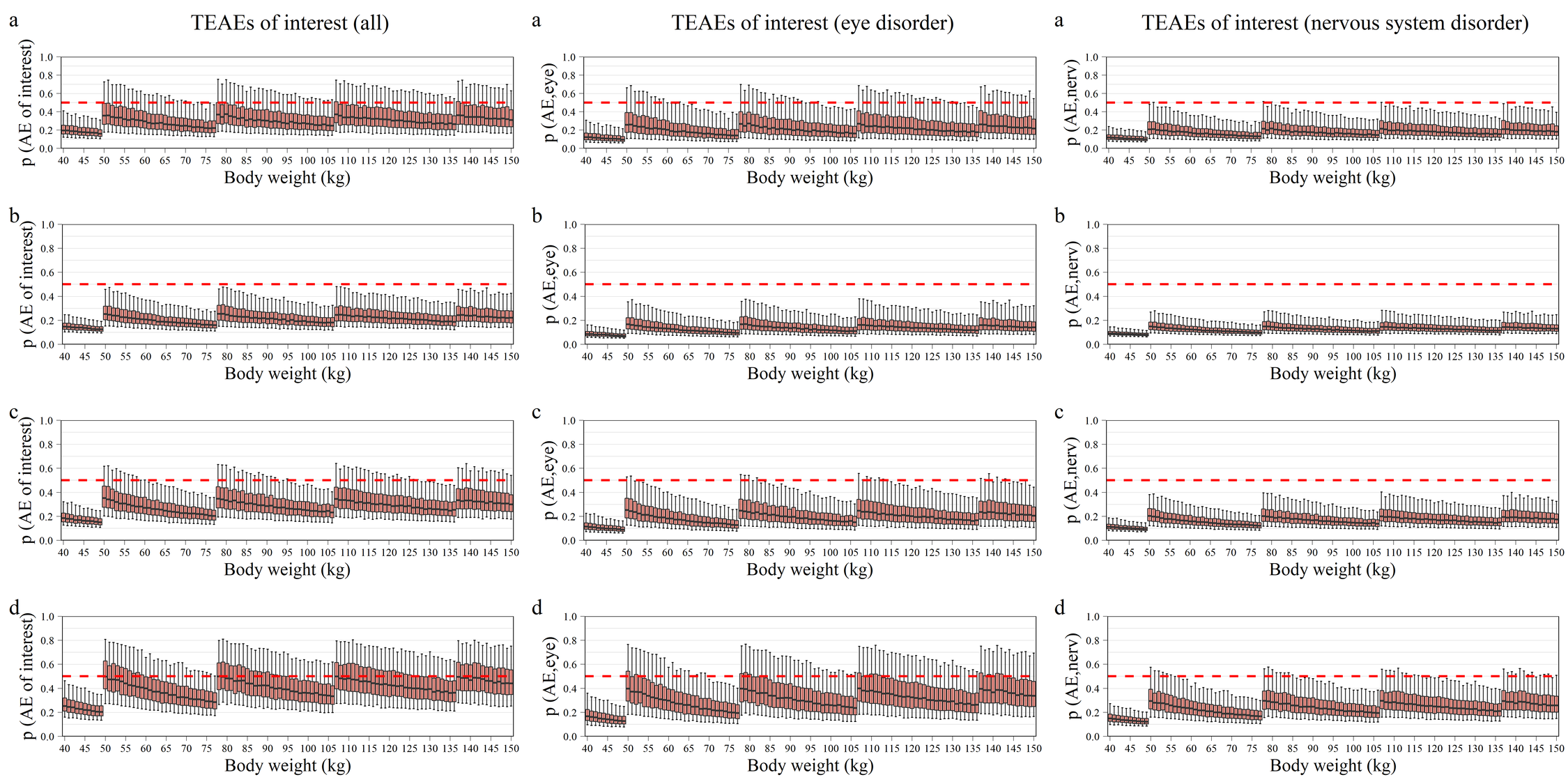

Supplement: S6 Fig — a: study arm 1 (single dose of emodespide), b: study arm 2 (once daily dosing for 7 days), c: study arm 3 (once daily dosing for 14 days) and d: study arm 4 (twice daily dosing for 10 days), administered in fasted state, as amorphous solid dispersion (ASD)-tablet B. Body weight-based dosing according to Fig 4 in the main text. The midline of the boxplots indicates the median, the box corresponds to the interquartile range, and the whiskers extend from the 5th to the 95th percentile. The red dotted line indicates a 50% probability threshold for any drug-related TEAE of interest. Probabilities of drug-related TEAE were predicted for all TEAE of interest (either eye disorder or nervous system disorder, left panel). Stratified analysis for eye disorders (middle panel) and nervous system disorders (right panel) are shown separately. (TIF) [file pntd.0010219.s013.tif]
