# Supplementary material for: Drug development for the treatment of onchocerciasis: Population pharmacokinetic and adverse events modeling of emodepside
Source: PLoS Negl Trop Dis. 2022 Mar 10;16(3):e0010219. doi: 10.1371/journal.pntd.0010219 (PMC8912909; doi:10.1371/journal.pntd.0010219)
Supplement: S1 Text — (DOCX) [file pntd.0010219.s014.docx]

**S1 Text**. **Rational for only including drug-related TEAEs in the logistic regression model for subsequent dose finding studies**

Non drug-related TEAEs were not included in the logistic regression model. There was no significant difference in the incidence of non-drug-related TEAEs between active treatment arms and placebo. A non-significant trend towards a higher incidence of non drug-related infections and infestations in the active treatment arms vs. placebo was observed (10.5% vs. 0%, p=0.13 (two-sided Fisher’s exact test)). However, infections and infestations occurred in only a few subjects (n=16) and a seasonal factor cannot be excluded. Onset of infections and infestations was later (most at >5 days post-dose) as compared to drug-related TEAEs of interest (most at 1–3 h post-dose). A logistic regression model to explore the relationship between the occurrence of infections and infestations and drug exposure (AUC_∞_) yielded weak model performance (Mc Fadden R^2^ = 0.09, area under the precision-recall curve: 29%), indicating that the model was not appropriate for the prediction of true positives. Therefore, only drug-related TEAEs of interest were included in the logistic regression model for subsequent dose finding studies.
