# Supplementary material for: Drug development for the treatment of onchocerciasis: Population pharmacokinetic and adverse events modeling of emodepside
Source: PLoS Negl Trop Dis. 2022 Mar 10;16(3):e0010219. doi: 10.1371/journal.pntd.0010219 (PMC8912909; doi:10.1371/journal.pntd.0010219)
Supplement: S1 Code — (DOCX) [file pntd.0010219.s015.docx]

**S1 Code**

NONMEM code of the final model

;; Modeler: Frauke Assmus, Richard Hoglund, Joel Tarning

;; Project name: Emodepside, Pooled Analysis

;;-------------------------------------------------------------------------------------------------------------;;

$SUBROUTINE ADVAN5 TRANS1

;;-------------------------------------------------------------------------------------------------------------;;

$MODEL

COMP=(1) ; Absorption compartment

COMP=(2) ; Central CMP

COMP=(3) ; Peripheral CMP1

COMP=(4) ; Peripheral CMP2

COMP=(5) ; Transit (1)

COMP=(6) ; Transit (2)

COMP=(7) ; Transit (3)

COMP=(8) ; Transit (4)

;;-------------------------------------------------------------------------------------------------------------;;

$PK

; Formulation effect

IF(FORM2.EQ.1) COV = 1 ; LSF solution

IF(FORM2.EQ.3) COV = 1+THETA(10) ; ASD-tablet formulation A

IF(FORM2.EQ.4) COV = 1+THETA(11) ; ASD-tablet formulation B

IF(FORM2.EQ.1) COV1 = 1

IF(FORM2.EQ.3) COV1 = 1+THETA(12)

IF(FORM2.EQ.4) COV1 = 1+THETA(13)

; Food effect

IF(FOOD.EQ.1) COV2 = 1 ; Fasted state

IF(FOOD.EQ.2) COV2 = 1+THETA(14) ; Fed state

IF(FOOD.EQ.1) COV3 = 1

IF(FOOD.EQ.2) COV3 = 1+THETA(15)

; Dose effect

COV4 = 1+THETA(16)*(DOSE_KG - 0.08)

; Inter-occasion variability

OCC1 = 0

OCC2 = 0

IF(OCC.EQ.1) OCC1=1 ; Day 0 – day 6 of dosing (MAD study), TAD < 144.1 h)

IF(OCC.EQ.2) OCC2=1 ; Day 7 – last day of dosing (MAD study), TAD ≥ 144.1h)

IOV = 0

IF(STUDY_ID.EQ.2) IOV = ETA(10)*OCC1+ETA(11)*OCC2

;;-------------------------------------------------------------------------------------------------------------;;

; Disposition parameters

TVCL = THETA(1)*((WT/75)**0.75) ; Elimination clearance

CL = TVCL*EXP(ETA(1))

TVV2 = THETA(2)*((WT/75)**1.00) ; Central volume

V2 = TVV2*EXP(ETA(2))

TVMT = THETA(3)*COV1 *COV2 * COV4 ; Mean transit time

MT = TVMT*EXP(ETA(3)+ IOV )

TVQ1 = THETA(4)*((WT/75)**0.75) ; Inter-compartment clearance (1)

Q1 = TVQ1*EXP(ETA(4))

TVV3 = THETA(5)*((WT/75)**1.00) ; Peripheral volume (1)

V3 = TVV3*EXP(ETA(5))

TVQ2 = THETA(6)*((WT/75)**0.75) ; Inter-compartment clearance (2)

Q2 = TVQ2*EXP(ETA(6))

TVV4 = THETA(7)*((WT/75)**1.00) ; Peripheral volume (2)

V4 = TVV4*EXP(ETA(7))

TVF1 = THETA(8) *COV *COV3 ; Relative bioavailability

F1 = TVF1*EXP(ETA(8))

TVCV = THETA(9) ; Plasma to DBS scaling

CV = TVCV*EXP(ETA(9))

NN = 4

KTR = (NN+1)/MT

K15 = KTR

K56 = KTR

K67 = KTR

K78 = KTR

K82 = KTR

K23 = Q1/V2

K32 = Q1/V3

K24 = Q2/V2

K42 = Q2/V4

K20 = CL/V2

S2 = V2

;;-------------------------------------------------------------------------------------------------------------;;

$ERROR

CPV = A(2)/S2 ; Predicted plasma concentration

CPC = CPV*CV ; Predicted capillary concentration

IF(MATRIX.EQ.1.AND.A(2).GT.0) IPRED=LOG(CPV) ; Plasma

IF(MATRIX.EQ.2.AND.A(2).GT.0) IPRED=LOG(CPC) ; Dry blood spot (capillary)

Y=IPRED+EPS(1)

IF(MATRIX.EQ.2) Y=IPRED+EPS(2)

W=SQRT(SIGMA(1,1))

IF(MATRIX.EQ.2) W=SQRT(SIGMA(2,2))

IRES = DV-IPRED

IWRES = IRES/W

IF(AMT.GT.0) DTIM=TIME

TAD=TIME-DTIM

;;-------------------------------------------------------------------------------------------------------------;;

$THETA

(0,1.29) ; 1 CL

(0,52.4) ; 2 V2

(0, 0.488) ; 3 MT

(0,8.45) ; 4 Q1

(0,647) ; 5 V3

(0,4.6) ; 6 Q2

(0,44.4) ; 7 V4

1 FIX ; 8 F1

(0,0.618) ; 9 CV

(-0.999,-0.314) ; 10 FORM2=3_F1

(-0.999,-0.2) ; 11 FORM2=4_F1

(-0.999,2.43) ; 12 FORM2=3_MT

(-0.999,1.24) ; 13 FORM2=4_MT

(-0.999,1.14) ; 14 FOOD_MT

(-0.999,-0.244) ; 15 FOOD_F1

(-1.695, 1.05,14.286) ; 16 DOSE_MT

;;-------------------------------------------------------------------------------------------------------------;;

$OMEGA

0.044 ; IIV_CL

0.0926 ; IIV_V2

0.135 ; IIV_MT

0.017 ; IIV_Q1

0.0908 ; IIV_V3

0.0843 ; IIV_Q2

0 FIX ; IIV_V4

0.0351 ; IIV_F1

0 FIX ; IIV_CV

$OMEGA BLOCK(1)

0.0692 ; IOV MT

$OMEGA BLOCK(1) SAME

;;-------------------------------------------------------------------------------------------------------------;;

$SIGMA

0.0203 ; Venous plasma data

0.0366 ; DBS data

;;-------------------------------------------------------------------------------------------------------------;;

$ESTIMATION

MAXEVAL=9999 PRINT=5 METHOD=1 INTER MCETA=50
